# Tris(η^5^-cyclo­penta­dien­yl)-tris­[η^6^-[9,10-dihydro­anthracene-9,10-*endo*-3′,4′-(*N*-benz­yl)pyrrolidine]]triruthenium(II) tris­(hexa­fluoro­phosphate) acetone disolvate

**DOI:** 10.1107/S1600536812040652

**Published:** 2012-09-29

**Authors:** Ielyzaveta Bratko, Sonia Mallet-Ladeira, Nathalie Saffon, Emmanuelle Teuma, Montserrat Gómez

**Affiliations:** a Laboratoire Hétérochimie Fondamentale et Appliquée, UMR CNRS 5069, Université Paul Sabatier, 118 route de Narbonne, 31062 Toulouse Cedex 9, France; b Institut de Chimie de Toulouse FR2599, UPS, Université de Toulouse, 118 route de Narbonne, 31062 Toulouse cedex 9, France

## Abstract

In the title compound, [Ru_3_(C_25_H_23_N)(C_5_H_5_)_3_]·3PF_6_·2C_3_H_6_O], the cation is a triruthenium complex of a 9,10-dihydro­anthracene derivative. Three RuCp^+^ (Cp is cyclo­penta­dien­yl) groups are bonded to the three aromatic rings of the ligand. Surprisingly, the pyramidalized N atom of the heterocycle (Σ C—N—C = 329.0°) points towards the anthracenyl group, so losing its coordinative ability. There is an inter­molecular C—H⋯π inter­action involving an acetone mol­ecule and the adjacent benzyl ring of the ligand. In the crystal, mol­ecules are linked *via* a number of C—H⋯O and C—H⋯F inter­actions and a C—H⋯π inter­action, leading to the formation of a three-dimensional supra­molecular structure. One of the Cp groups is disordered over two positions, with refined occupancies of 0.695 (14):0.305 (14). Two of the three hexa­fluoro­phospate anions are disordered, with refined occupancies of 0.630 (6):0.370 (6) and 0.771 (8):0.229 (8). One of the two solvent acetone mol­ecules is also disordered, with refined occupancies of 0.82 (2):0.18 (2).

## Related literature

For the synthesis and crystal structure of the free ligand, see: Bratko *et al.* (2012 ▶). For the synthesis of related ligands, see: Sanhes *et al.* (2008 ▶). For the coordination chemistry of related compounds with 9,10-dihydro­anthracene backbones fused with pyrrolidine groups, see: Sanhes *et al.* (2009 ▶, 2010 ▶).
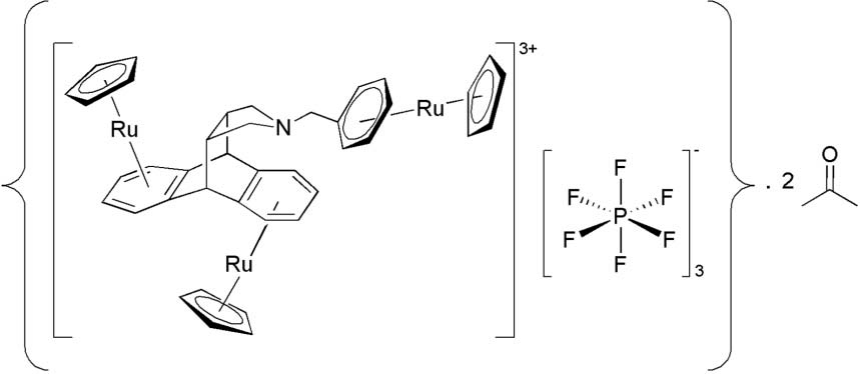


## Experimental

### 

#### Crystal data

[Ru_3_(C_25_H_23_N)(C_5_H_5_)_3_]·3PF_6_·2C_3_H_6_O]
*M* *_r_* = 1386.99
Monoclinic, 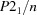

*a* = 9.6054 (7) Å
*b* = 36.567 (2) Å
*c* = 14.4207 (10) Å
β = 105.038 (3)°
*V* = 4891.6 (6) Å^3^
*Z* = 4
Mo *K*α radiation
μ = 1.12 mm^−1^
*T* = 193 K
0.16 × 0.02 × 0.02 mm

#### Data collection

Bruker Kappa APEXII CCD Quazar diffractometer
Absorption correction: multi-scan (*SADABS*; Bruker 2008 ▶) *T*_min_ = 0.841, *T*_max_ = 0.978
75190 measured reflections
11960 independent reflections
7646 reflections with *I* > 2σ(*I*)
*R*_int_ = 0.083

#### Refinement

*R*[*F* ^2^ > 2σ(*F* ^2^)] = 0.047
*wR*(*F* ^2^) = 0.093
*S* = 1.01
11960 reflections
871 parameters
738 restraints
H-atom parameters constrained
Δρ_max_ = 0.70 e Å^−3^
Δρ_min_ = −0.77 e Å^−3^

### 

Data collection: *APEX2* (Bruker, 2008 ▶); cell refinement: *APEX2* and *SAINT* (Bruker, 2008 ▶); data reduction: *SAINT*; program(s) used to solve structure: *SHELXS97* (Sheldrick, 2008 ▶); program(s) used to refine structure: *SHELXL97* (Sheldrick, 2008 ▶); molecular graphics: *SHELXTL* (Sheldrick, 2008 ▶); software used to prepare material for publication: *publCIF* (Westrip, 2010 ▶).

## Supplementary Material

Crystal structure: contains datablock(s) I, global. DOI: 10.1107/S1600536812040652/su2499sup1.cif

Structure factors: contains datablock(s) I. DOI: 10.1107/S1600536812040652/su2499Isup2.hkl

Additional supplementary materials:  crystallographic information; 3D view; checkCIF report

## Figures and Tables

**Table 1:** Hydrogen-bond geometry (Å, °) *Cg*1 is the centroid of the C20–C25 ring and *Cg*2 is the centroid of the C26–C30 ring.

*D*—H⋯*A*	*D*—H	H⋯*A*	*D*⋯*A*	*D*—H⋯*A*
C2—H2⋯O2^i^	0.95	2.56	3.188 (8)	124
C3—H3⋯O2^i^	0.95	2.58	3.197 (7)	123
C7—H7⋯F7^ii^	0.95	2.50	3.359 (5)	150
C8—H8⋯F9^iii^	0.95	2.42	3.302 (6)	155
C16—H16⋯O1	1.00	2.46	3.240 (6)	134
C18—H18*A*⋯F12^iii^	0.99	2.52	3.398 (5)	147
C22—H22⋯F11^iv^	0.95	2.43	3.091 (6)	127
C27—H27⋯O2^v^	0.95	2.50	3.141 (9)	125
C29—H29⋯F6^ii^	0.95	2.48	3.155 (7)	128
C43—H43*C*⋯F4^iv^	0.98	2.31	3.287 (9)	176
C46′—H46*D*⋯*Cg*1	0.98	2.79	3.63 (5)	160
C32—H32⋯*Cg*2^vi^	0.95	2.43	3.669 (5)	136

Symmetry codes: (i) 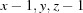; (ii) 
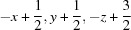
; (iii) 
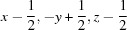
; (iv) 
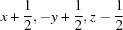
; (v) 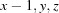; (vi) 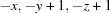.

**Fig. 1.**
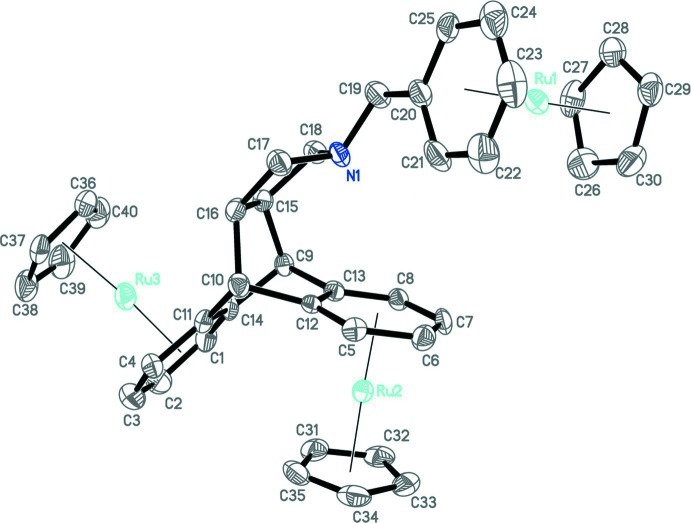
A view of the molecular structure of the title complex, with the atom numbering. Displacement ellipsoids are drawn at the 50% probability level. The three PF6- anions, the acteone solvent molecules, the minor fragments of the disordered atoms and the H atoms have been omitted for clarity.

**Fig. 2.**
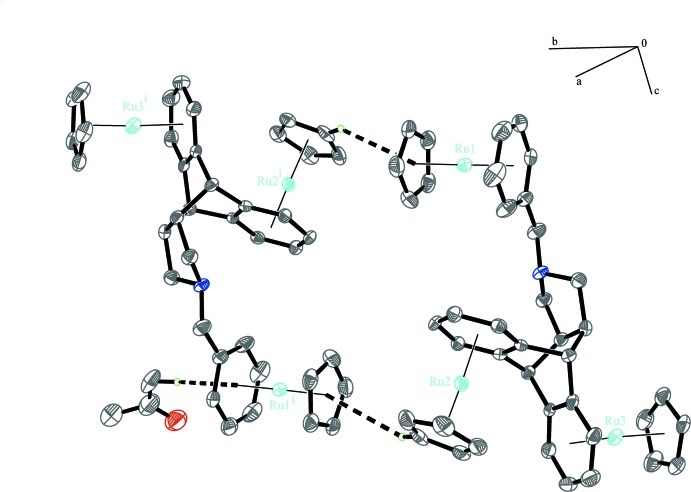
View showing the formation of the inversion dimer resulting from intermolecular C—H···π interactions, shown as dashed lines. [Symmetry code: (i) -x, 1 - y, 1 - z].

## supplementary crystallographic information

### Comment

In the last years, we have been interested in the design of new ligands for studying original ways to coordinate transition metals with the purpose of finding applications in homogeneous catalysis. Within this framework, we have explored the synthesis of original 9,10-dihydroanthracene backbones fused with pyrrolidine groups (Sanhes *et al.*, 2008). They offer the possibility of coordinating to metal atoms through both the heterocyclic moiety (Sanhes *et al.*, 2008, 2009, 2010) and the aromatic rings, leading to polymetallic systems potentially interesting for studying cooperative effects in catalysis. In contrast to the large number of works published concerning the π-coordination chemistry of anthracene and also 9,10-dihydroanthracene derivatives, no studies have been reported in relation to the π-interactions of Diels–Alder adducts involving anthracene with transition metals.

Our aim is to study the coordination chemistry of the new ligand 9,10-dihydroanthracene-9,10-*endo*-3',4'-(*N*-benzyl)pyrrolidine (Bratko *et al.*, 2012), through π-interactions with metal fragments. Ruthenium(II) organometallic precursors containing the RuCp^+^ moiety were chosen because of its known ability to give metal–arene complexes. Herein, we describe the crystal structure of, to the best of your knowledge, the first tris-ruthenium(II) complex of this kind of ligands.

The molecular structure of the title trinuclear ruthenium(II) cation is shown in Fig. 1. The asymmetric unit contains a tris(η^5^-cyclopentadienyl)-[(tris-η^6^)-(9,10-dihydroanthracene-9,10-*endo*-3',4'-(*N*-benzyl)-pyrrolidine)]-triruthenium(II) cation, three hexafluorophosphate anions and two molecules of acetone. In the cation, the N atom of the heterocycle is pyramidalized (Σ C—N—C = 328.95°), and points towards the anthracenyl group. This is in contrast to the situation in the free ligand (Bratko *et al.*, 2012), where the N atom is involved in a C—H···N interaction with the chloroform solvent molecule. In both compounds the pyrrolidine ring has an envelope conformation with the N atom as the flap. A weak intramolecular interaction between one H of the benzyl group (C21-H) and one of the aromatic rings of the backbone is observed (Fig. 1), favoured by the electron-deficient aromatic group of the 9,10-dihydroanthracenyl moiety upon ruthenium coordination. The deviation of atom N1 from the four planar atoms of the heterocycle in the ligand is 0.631 (1) Å, compared to 0.562 (3) Å in the title cation. There is a C—H···π interaction involving an acetone molecule and the adjacent benzyl ring of the ligand (Table 1).

In the crystal, molecules are linked via a second C—H···π interaction, leading to the formation of an inversion dimer (Table 1 and Fig. 2). There are also a number of C—H···O and C—H···F interactions present, resulting finally in the formation of a three-dimensional supramolecular structure (Table 1).

### Experimental

The trinuclear complex was prepared by adding [RuCp(CH_3_CN)_3_]PF_6_ (38.6 mg, 0.09 mmol) to a degassed solution of the ligand 9,10-dihydroanthracene-9,10-*endo*-3',4'-(*N*-benzyl)-pyrrolidine (10 mg, 0.03 mmol) in 8 ml of 1,2-dichloroethane. The resultant solution was then refluxed overnight. The reaction mixture was cooled and the solvent evaporated under vacuum. Single crystals were obtained from an acetone solution of the isolated complex at room temperature. The complex was characterized by high-resolution mass spectrometry (ES+, methanol): calc. mass for [C_40_H_38_NRu_3_.2PF_6_]^+ ^1126.9451; found: 1126.9471. Further spectroscopic data for the title compound are available in the archived CIF.

### Refinement

One of the cyclopentadyenyl groups (C36–C40/C36'–C40') is disordered over two positions with refined occupancies of 0.695 (14):0.305 (14). Two of the three hexafluorophospate anions (involving atoms P1 and P3) are disordered with refined occupancies of 0.630 (6):0.370 (6) and 0.771 (8):0.229 (8), respectively. One of the two molecules of solvent is also disordered with refined occupancies of 0.82 (2):0.18 (2). The SAME, DELU and SIMU restraints were applied for all of the disordered atoms (Sheldrick, 2008). All the H atoms were included in calulated positions and treated as riding: C—H = 0.95 Å (CH-aromatic), 0.98 Å (CH_3_), 0.99 Å (CH_2_) and 1.0 Å (CH-methine), with *Uiso*(H) = 1.5*Ueq*(C) for methyl H atoms and = 1.2*Ueq*(C) for other H atoms.

### Crystal data

**Table:**

[Ru_3_(C_25_H_23_N)(C_5_H_5_)_3_]·3PF_6_·2C_3_H_6_O]	*F*(000) = 2752
*M**_r_* = 1386.99	*D*_x_ = 1.883 Mg m^−^^3^
Monoclinic, *P*2_1_/*n*	Mo *K*α radiation, λ = 0.71073 Å
Hall symbol: -P 2yn	Cell parameters from 9276 reflections
*a* = 9.6054 (7) Å	θ = 2.5–23.9°
*b* = 36.567 (2) Å	µ = 1.12 mm^−^^1^
*c* = 14.4207 (10) Å	*T* = 193 K
β = 105.038 (3)°	Needle, colourless
*V* = 4891.6 (6) Å^3^	0.16 × 0.02 × 0.02 mm
*Z* = 4	

### Data collection

**Table:**

Bruker Kappa APEXII CCD Quazar diffractometer	11960 independent reflections
Radiation source: fine-focus sealed tube	7646 reflections with *I* > 2σ(*I*)
Graphite monochromator	*R*_int_ = 0.083
φ and ω scans	θ_max_ = 28.3°, θ_min_ = 2.7°
Absorption correction: multi-scan (*SADABS*; Bruker 2008)	*h* = −12→12
*T*_min_ = 0.841, *T*_max_ = 0.978	*k* = −46→48
75190 measured reflections	*l* = −18→19

### Refinement

**Table:**

Refinement on *F*^2^	Primary atom site location: structure-invariant direct methods
Least-squares matrix: full	Secondary atom site location: difference Fourier map
*R*[*F*^2^ > 2σ(*F*^2^)] = 0.047	Hydrogen site location: inferred from neighbouring sites
*wR*(*F*^2^) = 0.093	H-atom parameters constrained
*S* = 1.01	*w* = 1/[σ^2^(*F*_o_^2^) + (0.0298*P*)^2^ + 6.328*P*] where *P* = (*F*_o_^2^ + 2*F*_c_^2^)/3
11960 reflections	(Δ/σ)_max_ = 0.002
871 parameters	Δρ_max_ = 0.70 e Å^−^^3^
738 restraints	Δρ_min_ = −0.77 e Å^−^^3^

### Special details

**Table:**

Experimental. Spectroscopic data for the title compound: ^1^H NMR (300 MHz in (CD_3_)_2_CO): 6.68–6.70 (m, 2H, Harom); 6.56–6.58 (m, 2H, Harom); 6.35–6.37 (m, 2H, Harom); 6.30–6.31 (m, 3H, Harom of Bn); 6.23–6.26 (m, 2H, Harom); 6.00–6.03 (m, 2H, Harom); 5.67 (m, 5H, Cp); 5.43 (s, 5H,Cp); 4.91 (s, 5H, Cp); 4.31 (m, 2H, H_9,10_); 3.16 (s, 2H, H_19_); 3.08–3.10 (m, 2H, H_17_ or H_18_); 2.78–2.84 (m, 2H, H_15+16_); 2.49–2.54 (m, 2H, H_17_ or H_18_). ^13^C NMR (75 MHz in (CD_3_)_2_CO): 115.3, 107.0 (C_11,12,13,14_); 87.5 (CHarom); 87.3 (CH of Bn); 87.1, 85.9, 84.9, 84.7 (CH arom); 83.2 (Cp); 82.5(Cp); 82.3 (Cp); 58.7 (C_19_); 58.5 (C_17,18_); 48.2 (C_15,16_); 44.3(C_9,10_).
Geometry. Bond distances, angles etc. have been calculated using the rounded fractional coordinates. All su's are estimated from the variances of the (full) variance-covariance matrix. The cell esds are taken into account in the estimation of distances, angles and torsion angles
Refinement. Refinement of *F*^2^ against ALL reflections. The weighted *R*-factor *wR* and goodness of fit *S* are based on *F*^2^, conventional *R*-factors *R* are based on *F*, with *F* set to zero for negative *F*^2^. The threshold expression of *F*^2^ > σ(*F*^2^) is used only for calculating *R*-factors(gt) *etc*. and is not relevant to the choice of reflections for refinement. *R*-factors based on *F*^2^ are statistically about twice as large as those based on *F*, and *R*-factors based on ALL data will be even larger.

### Fractional atomic coordinates and isotropic or equivalent isotropic displacement parameters (Å^2^)

**Table:**

	*x*	*y*	*z*	*U*_iso_*/*U*_eq_	Occ. (<1)
Ru1	0.33854 (4)	0.42603 (1)	0.72728 (2)	0.0236 (1)	
Ru2	0.16059 (4)	0.45640 (1)	0.22459 (2)	0.0216 (1)	
Ru3	0.10104 (4)	0.31457 (1)	0.07175 (3)	0.0299 (1)	
N1	0.2080 (4)	0.36051 (9)	0.4696 (2)	0.0237 (11)	
C1	−0.0441 (5)	0.36216 (11)	0.0595 (3)	0.0320 (16)	
C2	0.0094 (6)	0.35921 (12)	−0.0230 (3)	0.0376 (16)	
C3	0.1566 (6)	0.35806 (12)	−0.0146 (3)	0.0389 (19)	
C4	0.2558 (5)	0.35895 (11)	0.0760 (3)	0.0291 (14)	
C5	0.3412 (4)	0.43265 (10)	0.3366 (3)	0.0219 (12)	
C6	0.2829 (5)	0.46293 (11)	0.3753 (3)	0.0266 (14)	
C7	0.1353 (5)	0.46418 (11)	0.3708 (3)	0.0272 (14)	
C8	0.0416 (5)	0.43567 (10)	0.3259 (3)	0.0222 (12)	
C9	0.0203 (4)	0.37137 (10)	0.2446 (3)	0.0199 (12)	
C10	0.2952 (5)	0.36794 (10)	0.2607 (3)	0.0231 (12)	
C11	0.2045 (5)	0.36303 (10)	0.1584 (3)	0.0239 (14)	
C12	0.2499 (4)	0.40405 (10)	0.2957 (3)	0.0177 (11)	
C13	0.1009 (4)	0.40561 (10)	0.2885 (3)	0.0198 (12)	
C14	0.0552 (4)	0.36497 (10)	0.1504 (3)	0.0215 (12)	
C15	0.0868 (4)	0.33976 (10)	0.3140 (3)	0.0222 (12)	
C16	0.2525 (4)	0.33798 (10)	0.3246 (3)	0.0230 (12)	
C17	0.3190 (5)	0.34224 (12)	0.4334 (3)	0.0274 (12)	
C18	0.0719 (5)	0.34417 (11)	0.4153 (3)	0.0270 (14)	
C19	0.2248 (5)	0.35578 (12)	0.5717 (3)	0.0324 (14)	
C20	0.3463 (5)	0.37695 (11)	0.6362 (3)	0.0286 (14)	
C21	0.4125 (5)	0.40690 (12)	0.6039 (3)	0.0362 (16)	
C22	0.5248 (5)	0.42606 (14)	0.6686 (4)	0.0442 (17)	
C23	0.5708 (5)	0.41519 (15)	0.7654 (4)	0.0444 (17)	
C24	0.5040 (5)	0.38608 (13)	0.7984 (4)	0.0418 (17)	
C25	0.3914 (5)	0.36709 (11)	0.7339 (3)	0.0315 (16)	
C26	0.1629 (6)	0.46440 (13)	0.6732 (4)	0.0441 (17)	
C27	0.1147 (5)	0.43607 (12)	0.7228 (3)	0.0363 (17)	
C28	0.1983 (5)	0.43684 (12)	0.8190 (3)	0.0352 (17)	
C29	0.2988 (5)	0.46588 (12)	0.8285 (3)	0.0344 (17)	
C30	0.2756 (6)	0.48300 (12)	0.7385 (4)	0.0408 (16)	
C31	0.0434 (5)	0.45710 (12)	0.0730 (3)	0.0352 (14)	
C32	0.0054 (5)	0.48785 (12)	0.1192 (3)	0.0343 (17)	
C33	0.1292 (5)	0.50943 (12)	0.1553 (3)	0.0363 (16)	
C34	0.2460 (5)	0.49184 (13)	0.1307 (3)	0.0382 (17)	
C35	0.1924 (5)	0.45915 (13)	0.0803 (3)	0.0357 (16)	
C36	0.1527 (15)	0.2641 (2)	0.1538 (5)	0.035 (2)	0.695 (14)
C37	0.2299 (8)	0.2650 (2)	0.0824 (9)	0.034 (2)	0.695 (14)
C38	0.1291 (13)	0.2664 (3)	−0.0090 (6)	0.040 (2)	0.695 (14)
C39	−0.0087 (10)	0.2656 (3)	0.0066 (8)	0.045 (2)	0.695 (14)
C40	0.0047 (12)	0.2642 (2)	0.1068 (10)	0.041 (2)	0.695 (14)
C36'	0.080 (4)	0.2637 (6)	0.1490 (12)	0.038 (3)	0.305 (14)
C37'	0.210 (2)	0.2651 (6)	0.120 (2)	0.038 (3)	0.305 (14)
C38'	0.174 (3)	0.2642 (7)	0.0184 (19)	0.039 (3)	0.305 (14)
C39'	0.023 (3)	0.2653 (9)	−0.0137 (13)	0.040 (3)	0.305 (14)
C40'	−0.036 (2)	0.2645 (6)	0.066 (2)	0.038 (3)	0.305 (14)
P1	0.1054 (5)	0.07542 (11)	0.5810 (3)	0.0289 (8)	0.771 (8)
F1	0.2519 (7)	0.0632 (2)	0.6539 (6)	0.056 (2)	0.771 (8)
F2	−0.0407 (7)	0.0877 (2)	0.5070 (6)	0.0428 (19)	0.771 (8)
F3	0.0238 (5)	0.0703 (3)	0.6613 (3)	0.079 (2)	0.771 (8)
F4	0.1375 (6)	0.11660 (12)	0.6126 (5)	0.0693 (18)	0.771 (8)
F5	0.1878 (5)	0.08150 (19)	0.4999 (3)	0.0600 (18)	0.771 (8)
F6	0.0749 (6)	0.03495 (11)	0.5467 (5)	0.083 (2)	0.771 (8)
P2	0.26055 (12)	0.06345 (3)	0.98701 (8)	0.0278 (3)	
F7	0.3738 (3)	0.03292 (7)	0.97633 (19)	0.0480 (10)	
F8	0.1462 (3)	0.09362 (7)	0.9970 (2)	0.0483 (10)	
F9	0.2260 (3)	0.07187 (8)	0.87556 (18)	0.0502 (10)	
F10	0.1356 (3)	0.03332 (7)	0.9670 (2)	0.0520 (10)	
F11	0.2941 (3)	0.05429 (7)	1.09911 (17)	0.0412 (9)	
F12	0.3842 (3)	0.09325 (8)	1.0084 (2)	0.0572 (11)	
P3	0.0304 (7)	0.22339 (17)	0.3995 (4)	0.0452 (12)	0.630 (6)
F13	−0.0908 (10)	0.1947 (3)	0.4051 (7)	0.086 (3)	0.630 (6)
F14	0.1504 (11)	0.2524 (3)	0.3901 (8)	0.066 (3)	0.630 (6)
F15	0.1541 (8)	0.1989 (2)	0.4607 (7)	0.103 (3)	0.630 (6)
F16	0.0423 (11)	0.20427 (19)	0.3037 (5)	0.089 (3)	0.630 (6)
F17	−0.0885 (8)	0.2497 (2)	0.3388 (6)	0.102 (3)	0.630 (6)
F18	0.0114 (10)	0.24368 (18)	0.4908 (5)	0.085 (3)	0.630 (6)
P1'	0.098 (2)	0.0765 (4)	0.5813 (12)	0.039 (2)	0.229 (8)
F1'	0.254 (2)	0.0743 (7)	0.6533 (19)	0.048 (5)	0.229 (8)
F2'	−0.058 (2)	0.0803 (8)	0.508 (2)	0.039 (4)	0.229 (8)
F3'	0.037 (2)	0.0938 (6)	0.6624 (10)	0.056 (4)	0.229 (8)
F4'	0.1324 (18)	0.1166 (4)	0.5543 (14)	0.061 (4)	0.229 (8)
F5'	0.154 (2)	0.0604 (6)	0.4957 (11)	0.062 (4)	0.229 (8)
F6'	0.0532 (18)	0.0382 (4)	0.6097 (16)	0.068 (4)	0.229 (8)
P3'	0.0318 (14)	0.2223 (4)	0.4008 (9)	0.063 (2)	0.370 (6)
F13'	−0.0574 (19)	0.1919 (5)	0.4378 (13)	0.090 (4)	0.370 (6)
F14'	0.125 (2)	0.2533 (6)	0.3677 (15)	0.075 (4)	0.370 (6)
F15'	0.1375 (13)	0.1921 (3)	0.3847 (12)	0.086 (3)	0.370 (6)
F16'	−0.0595 (16)	0.2184 (3)	0.2931 (8)	0.092 (3)	0.370 (6)
F17'	−0.0751 (13)	0.2529 (3)	0.4159 (12)	0.088 (4)	0.370 (6)
F18'	0.1194 (17)	0.2283 (4)	0.5071 (7)	0.102 (4)	0.370 (6)
O1	0.5020 (4)	0.28486 (11)	0.2867 (3)	0.0702 (18)	
C41	0.6113 (6)	0.30171 (15)	0.2912 (5)	0.055 (2)	
C42	0.6821 (7)	0.32293 (19)	0.3779 (6)	0.096 (3)	
C43	0.6812 (8)	0.3018 (2)	0.2116 (5)	0.099 (4)	
O2	1.0209 (13)	0.35608 (19)	0.7582 (4)	0.075 (3)	0.82 (2)
C44	0.9494 (13)	0.3322 (2)	0.7141 (6)	0.053 (3)	0.82 (2)
C45	1.0180 (13)	0.2975 (3)	0.6967 (10)	0.097 (5)	0.82 (2)
C46	0.7926 (14)	0.3362 (3)	0.6691 (11)	0.106 (6)	0.82 (2)
O2'	0.925 (5)	0.3647 (7)	0.7532 (18)	0.065 (6)	0.18 (2)
C44'	0.880 (5)	0.3379 (10)	0.708 (4)	0.066 (7)	0.18 (2)
C45'	0.975 (5)	0.3050 (11)	0.723 (3)	0.073 (8)	0.18 (2)
C46'	0.727 (5)	0.3335 (12)	0.657 (3)	0.062 (8)	0.18 (2)
H6	0.34400	0.48250	0.40430	0.0320*	
H7	0.09770	0.48430	0.39820	0.0330*	
H1	−0.14490	0.36220	0.05370	0.0380*	
H2	−0.05620	0.35800	−0.08480	0.0450*	
H3	0.19050	0.35670	−0.07080	0.0470*	
H4	0.35630	0.35680	0.08160	0.0350*	
H5	0.44070	0.43190	0.33850	0.0260*	
H17A	0.34300	0.31810	0.46420	0.0330*	
H17B	0.40780	0.35720	0.44600	0.0330*	
H18A	−0.01030	0.36030	0.41630	0.0320*	
H18B	0.05650	0.32020	0.44280	0.0320*	
H19A	0.24000	0.32950	0.58710	0.0390*	
H19B	0.13360	0.36310	0.58650	0.0390*	
H21	0.38180	0.41430	0.53870	0.0440*	
H22	0.56930	0.44630	0.64660	0.0530*	
H23	0.64770	0.42780	0.80820	0.0530*	
H24	0.53380	0.37900	0.86400	0.0500*	
H25	0.34530	0.34730	0.75670	0.0380*	
H26	0.12610	0.47010	0.60700	0.0530*	
H27	0.03910	0.41940	0.69630	0.0440*	
H28	0.18880	0.42070	0.86870	0.0420*	
H29	0.36910	0.47260	0.88550	0.0420*	
H30	0.32700	0.50350	0.72410	0.0490*	
H31	−0.02000	0.43830	0.04220	0.0420*	
H32	−0.08860	0.49330	0.12520	0.0410*	
H33	0.13350	0.53170	0.18980	0.0440*	
H34	0.34280	0.50030	0.14530	0.0460*	
H35	0.24730	0.44180	0.05600	0.0430*	
H36	0.19350	0.26350	0.22120	0.0420*	0.695 (14)
H37	0.33190	0.26460	0.09380	0.0410*	0.695 (14)
H38	0.15110	0.26770	−0.06950	0.0470*	0.695 (14)
H39	−0.09700	0.26600	−0.04210	0.0540*	0.695 (14)
H40	−0.07240	0.26350	0.13690	0.0490*	0.695 (14)
H8	−0.05880	0.43680	0.32110	0.0270*	
H9	−0.08590	0.37370	0.23680	0.0240*	
H10	0.40070	0.36740	0.26470	0.0270*	
H15	0.04180	0.31610	0.28700	0.0260*	
H16	0.27790	0.31340	0.30350	0.0280*	
H36'	0.07210	0.26240	0.21330	0.0450*	0.305 (14)
H37'	0.30430	0.26640	0.16110	0.0460*	0.305 (14)
H38'	0.23950	0.26310	−0.02070	0.0470*	0.305 (14)
H39'	−0.03080	0.26630	−0.07900	0.0480*	0.305 (14)
H40'	−0.13550	0.26450	0.06440	0.0460*	0.305 (14)
H42A	0.62380	0.32160	0.42440	0.1440*	
H42B	0.69140	0.34850	0.36010	0.1440*	
H42C	0.77800	0.31270	0.40660	0.1440*	
H43A	0.63910	0.28250	0.16570	0.1490*	
H43B	0.78480	0.29730	0.23690	0.1490*	
H43C	0.66640	0.32560	0.17930	0.1490*	
H45A	0.99740	0.29310	0.62740	0.1460*	0.82 (2)
H45B	0.97950	0.27730	0.72720	0.1460*	0.82 (2)
H45C	1.12250	0.29920	0.72380	0.1460*	0.82 (2)
H46A	0.74320	0.34260	0.71840	0.1590*	0.82 (2)
H46B	0.75410	0.31300	0.63880	0.1590*	0.82 (2)
H46C	0.77680	0.35550	0.62040	0.1590*	0.82 (2)
H45D	1.03370	0.30520	0.67630	0.1090*	0.18 (2)
H45E	0.91550	0.28290	0.71350	0.1090*	0.18 (2)
H45F	1.03850	0.30520	0.78810	0.1090*	0.18 (2)
H46D	0.67310	0.35530	0.66760	0.0930*	0.18 (2)
H46E	0.68770	0.31190	0.68220	0.0930*	0.18 (2)
H46F	0.71730	0.33040	0.58850	0.0930*	0.18 (2)

### Atomic displacement parameters (Å^2^)

**Table:**

	*U*^11^	*U*^22^	*U*^33^	*U*^12^	*U*^13^	*U*^23^
Ru1	0.0267 (2)	0.0254 (2)	0.0182 (2)	0.0005 (2)	0.0047 (1)	−0.0010 (1)
Ru2	0.0226 (2)	0.0200 (2)	0.0229 (2)	0.0027 (1)	0.0073 (1)	−0.0001 (1)
Ru3	0.0363 (2)	0.0283 (2)	0.0252 (2)	0.0004 (2)	0.0082 (2)	−0.0074 (2)
N1	0.028 (2)	0.0276 (18)	0.0170 (17)	−0.0015 (16)	0.0086 (15)	−0.0051 (14)
C1	0.032 (3)	0.030 (2)	0.030 (3)	0.007 (2)	0.001 (2)	−0.0070 (19)
C2	0.053 (3)	0.035 (3)	0.021 (2)	0.004 (2)	0.003 (2)	0.0002 (19)
C3	0.055 (4)	0.037 (3)	0.027 (3)	−0.001 (2)	0.015 (2)	−0.002 (2)
C4	0.036 (3)	0.026 (2)	0.030 (2)	0.001 (2)	0.017 (2)	−0.0024 (18)
C5	0.019 (2)	0.024 (2)	0.021 (2)	−0.0011 (17)	0.0021 (17)	0.0009 (17)
C6	0.030 (3)	0.022 (2)	0.026 (2)	−0.0011 (18)	0.0043 (19)	−0.0021 (17)
C7	0.039 (3)	0.025 (2)	0.019 (2)	0.0076 (19)	0.010 (2)	−0.0058 (17)
C8	0.024 (2)	0.023 (2)	0.021 (2)	0.0038 (17)	0.0083 (18)	0.0000 (16)
C9	0.017 (2)	0.022 (2)	0.020 (2)	−0.0017 (17)	0.0035 (17)	−0.0045 (16)
C10	0.025 (2)	0.024 (2)	0.019 (2)	0.0016 (18)	0.0034 (18)	−0.0026 (17)
C11	0.032 (3)	0.016 (2)	0.024 (2)	0.0007 (18)	0.008 (2)	−0.0039 (16)
C12	0.022 (2)	0.018 (2)	0.0130 (19)	0.0026 (16)	0.0043 (17)	0.0024 (15)
C13	0.022 (2)	0.022 (2)	0.014 (2)	0.0015 (17)	0.0020 (17)	0.0009 (16)
C14	0.025 (2)	0.018 (2)	0.020 (2)	0.0052 (17)	0.0031 (18)	−0.0040 (16)
C15	0.027 (2)	0.019 (2)	0.020 (2)	−0.0035 (17)	0.0053 (18)	−0.0034 (16)
C16	0.026 (2)	0.018 (2)	0.025 (2)	0.0018 (17)	0.0066 (19)	−0.0016 (17)
C17	0.027 (2)	0.031 (2)	0.025 (2)	0.0036 (19)	0.0083 (19)	0.0004 (18)
C18	0.032 (3)	0.027 (2)	0.024 (2)	−0.0031 (19)	0.011 (2)	−0.0006 (18)
C19	0.041 (3)	0.033 (2)	0.026 (2)	−0.009 (2)	0.014 (2)	−0.0046 (19)
C20	0.033 (3)	0.032 (2)	0.023 (2)	0.003 (2)	0.011 (2)	−0.0035 (18)
C21	0.045 (3)	0.044 (3)	0.026 (2)	−0.007 (2)	0.021 (2)	−0.008 (2)
C22	0.040 (3)	0.048 (3)	0.054 (3)	−0.014 (2)	0.029 (3)	−0.013 (3)
C23	0.027 (3)	0.057 (3)	0.046 (3)	0.002 (2)	0.004 (2)	−0.022 (3)
C24	0.040 (3)	0.045 (3)	0.036 (3)	0.021 (3)	0.002 (2)	−0.008 (2)
C25	0.039 (3)	0.025 (2)	0.030 (3)	0.011 (2)	0.008 (2)	−0.0041 (19)
C26	0.047 (3)	0.044 (3)	0.037 (3)	0.020 (3)	0.003 (3)	0.005 (2)
C27	0.023 (3)	0.033 (3)	0.051 (3)	0.006 (2)	0.006 (2)	−0.013 (2)
C28	0.041 (3)	0.033 (3)	0.037 (3)	0.009 (2)	0.020 (2)	−0.001 (2)
C29	0.035 (3)	0.031 (3)	0.036 (3)	0.010 (2)	0.007 (2)	−0.007 (2)
C30	0.049 (3)	0.025 (2)	0.049 (3)	0.004 (2)	0.014 (3)	0.000 (2)
C31	0.050 (3)	0.028 (2)	0.024 (2)	0.002 (2)	0.003 (2)	0.0081 (19)
C32	0.033 (3)	0.037 (3)	0.033 (3)	0.012 (2)	0.009 (2)	0.018 (2)
C33	0.046 (3)	0.025 (2)	0.041 (3)	0.006 (2)	0.017 (2)	0.009 (2)
C34	0.039 (3)	0.041 (3)	0.040 (3)	−0.001 (2)	0.020 (2)	0.015 (2)
C35	0.044 (3)	0.042 (3)	0.027 (2)	0.014 (2)	0.020 (2)	0.009 (2)
C36	0.050 (5)	0.021 (3)	0.035 (3)	−0.002 (4)	0.015 (3)	−0.005 (3)
C37	0.046 (4)	0.022 (3)	0.036 (4)	0.005 (3)	0.013 (3)	−0.008 (3)
C38	0.050 (5)	0.034 (3)	0.032 (4)	0.003 (4)	0.006 (3)	−0.013 (3)
C39	0.046 (4)	0.039 (3)	0.047 (4)	−0.009 (4)	0.009 (4)	−0.018 (4)
C40	0.046 (4)	0.030 (3)	0.047 (5)	−0.007 (4)	0.014 (4)	−0.012 (4)
C36'	0.050 (6)	0.026 (5)	0.038 (5)	−0.003 (6)	0.013 (5)	−0.007 (5)
C37'	0.048 (5)	0.027 (5)	0.039 (6)	0.002 (5)	0.010 (5)	−0.008 (5)
C38'	0.048 (5)	0.030 (5)	0.040 (5)	0.001 (5)	0.014 (5)	−0.013 (5)
C39'	0.050 (6)	0.036 (5)	0.035 (5)	−0.008 (5)	0.015 (5)	−0.018 (5)
C40'	0.046 (5)	0.032 (5)	0.037 (6)	−0.008 (5)	0.014 (5)	−0.012 (6)
P1	0.0246 (14)	0.0324 (13)	0.0265 (13)	−0.0016 (12)	0.0008 (11)	0.0006 (12)
F1	0.038 (3)	0.074 (5)	0.045 (3)	0.011 (3)	−0.011 (2)	0.008 (3)
F2	0.028 (3)	0.058 (4)	0.036 (3)	0.003 (2)	−0.003 (2)	0.006 (3)
F3	0.046 (3)	0.155 (6)	0.033 (2)	−0.024 (4)	0.0078 (19)	0.021 (3)
F4	0.064 (3)	0.049 (2)	0.082 (4)	−0.003 (2)	−0.004 (3)	−0.028 (3)
F5	0.041 (3)	0.104 (4)	0.038 (2)	0.001 (3)	0.0158 (19)	0.012 (3)
F6	0.104 (4)	0.027 (2)	0.086 (4)	−0.003 (2)	−0.031 (3)	−0.002 (2)
P2	0.0248 (6)	0.0338 (6)	0.0255 (6)	0.0025 (5)	0.0078 (5)	0.0078 (5)
F7	0.0492 (18)	0.0548 (18)	0.0438 (17)	0.0218 (14)	0.0188 (14)	0.0107 (14)
F8	0.0517 (19)	0.0462 (17)	0.0535 (18)	0.0212 (14)	0.0255 (15)	0.0119 (14)
F9	0.0493 (19)	0.077 (2)	0.0269 (14)	0.0233 (16)	0.0144 (13)	0.0212 (14)
F10	0.0394 (17)	0.0513 (18)	0.0562 (19)	−0.0142 (14)	−0.0039 (15)	0.0106 (14)
F11	0.0344 (16)	0.0637 (18)	0.0249 (14)	0.0029 (13)	0.0065 (12)	0.0096 (12)
F12	0.0465 (19)	0.0540 (18)	0.069 (2)	−0.0229 (15)	0.0113 (16)	0.0123 (15)
P3	0.058 (2)	0.032 (2)	0.052 (2)	−0.0043 (18)	0.0259 (19)	−0.0014 (19)
F13	0.099 (5)	0.085 (5)	0.090 (6)	−0.043 (4)	0.056 (5)	−0.023 (4)
F14	0.071 (4)	0.051 (4)	0.075 (6)	−0.020 (3)	0.016 (4)	0.002 (4)
F15	0.097 (4)	0.079 (4)	0.128 (6)	0.022 (4)	0.019 (4)	0.050 (4)
F16	0.133 (6)	0.067 (4)	0.087 (4)	−0.041 (4)	0.066 (4)	−0.033 (3)
F17	0.091 (4)	0.100 (5)	0.096 (5)	0.025 (4)	−0.010 (4)	0.013 (4)
F18	0.123 (6)	0.082 (4)	0.060 (4)	−0.007 (4)	0.043 (4)	−0.022 (3)
P1'	0.035 (4)	0.045 (4)	0.034 (4)	0.000 (4)	0.005 (4)	0.001 (4)
F1'	0.034 (6)	0.063 (10)	0.038 (7)	0.004 (6)	−0.004 (5)	0.008 (7)
F2'	0.029 (6)	0.043 (8)	0.038 (7)	−0.008 (6)	−0.002 (6)	−0.001 (6)
F3'	0.052 (7)	0.082 (8)	0.030 (6)	0.024 (7)	0.006 (5)	−0.010 (6)
F4'	0.054 (7)	0.054 (5)	0.062 (8)	−0.028 (5)	−0.010 (7)	0.019 (6)
F5'	0.052 (7)	0.090 (8)	0.042 (6)	0.025 (7)	0.011 (5)	−0.010 (6)
F6'	0.068 (7)	0.043 (6)	0.076 (8)	−0.024 (5)	−0.012 (7)	0.025 (6)
P3'	0.075 (4)	0.048 (4)	0.067 (4)	−0.010 (4)	0.022 (4)	−0.005 (4)
F13'	0.103 (8)	0.069 (6)	0.101 (9)	−0.023 (5)	0.031 (7)	0.023 (6)
F14'	0.099 (8)	0.052 (6)	0.079 (8)	−0.026 (6)	0.033 (7)	−0.004 (6)
F15'	0.092 (6)	0.056 (5)	0.112 (7)	0.007 (4)	0.032 (6)	−0.012 (6)
F16'	0.111 (7)	0.067 (6)	0.076 (5)	−0.015 (6)	−0.014 (5)	−0.001 (5)
F17'	0.094 (6)	0.063 (5)	0.114 (8)	0.007 (5)	0.040 (6)	−0.011 (6)
F18'	0.122 (8)	0.105 (7)	0.067 (5)	−0.009 (6)	0.003 (5)	−0.005 (5)
O1	0.040 (2)	0.068 (3)	0.105 (4)	−0.008 (2)	0.023 (2)	−0.015 (2)
C41	0.042 (4)	0.040 (3)	0.078 (4)	0.010 (3)	0.007 (3)	0.007 (3)
C42	0.050 (4)	0.097 (5)	0.132 (7)	−0.002 (4)	0.008 (4)	−0.061 (5)
C43	0.085 (6)	0.127 (7)	0.089 (6)	−0.015 (5)	0.029 (5)	0.029 (5)
O2	0.107 (7)	0.073 (4)	0.045 (3)	−0.032 (4)	0.018 (3)	−0.008 (3)
C44	0.083 (7)	0.044 (5)	0.033 (4)	−0.023 (5)	0.019 (5)	0.003 (3)
C45	0.108 (9)	0.065 (6)	0.131 (10)	−0.035 (6)	0.054 (7)	−0.010 (6)
C46	0.087 (10)	0.074 (7)	0.146 (12)	−0.009 (7)	0.012 (10)	0.022 (7)
O2'	0.080 (12)	0.060 (9)	0.055 (9)	−0.011 (8)	0.017 (8)	−0.010 (7)
C44'	0.082 (14)	0.059 (11)	0.059 (11)	−0.007 (10)	0.024 (10)	−0.016 (9)
C45'	0.084 (16)	0.067 (13)	0.066 (14)	−0.002 (12)	0.017 (13)	−0.014 (12)
C46'	0.079 (16)	0.052 (13)	0.061 (13)	−0.005 (12)	0.030 (12)	−0.029 (11)

### Geometric parameters (Å, º)

**Table:**

Ru1—C20	2.237 (4)	C19—C20	1.504 (6)
Ru1—C21	2.195 (4)	C20—C25	1.409 (6)
Ru1—C22	2.169 (5)	C20—C21	1.405 (6)
Ru1—C23	2.191 (5)	C21—C22	1.415 (7)
Ru1—C24	2.206 (5)	C22—C23	1.408 (8)
Ru1—C25	2.211 (4)	C23—C24	1.389 (7)
Ru1—C26	2.177 (5)	C24—C25	1.413 (7)
Ru1—C27	2.166 (5)	C26—C30	1.412 (8)
Ru1—C28	2.155 (5)	C26—C27	1.404 (7)
Ru1—C29	2.165 (4)	C27—C28	1.411 (6)
Ru1—C30	2.187 (5)	C28—C29	1.418 (7)
Ru2—C5	2.218 (4)	C29—C30	1.406 (7)
Ru2—C6	2.199 (4)	C31—C35	1.410 (7)
Ru2—C7	2.203 (4)	C31—C32	1.403 (6)
Ru2—C8	2.210 (5)	C32—C33	1.409 (7)
Ru2—C12	2.235 (4)	C33—C34	1.416 (7)
Ru2—C13	2.214 (4)	C34—C35	1.424 (7)
Ru2—C31	2.184 (4)	C36—C37	1.417 (16)
Ru2—C32	2.163 (4)	C36—C40	1.407 (18)
Ru2—C33	2.167 (4)	C36'—C37'	1.42 (4)
Ru2—C34	2.182 (5)	C36'—C40'	1.41 (4)
Ru2—C35	2.182 (4)	C37—C38	1.420 (15)
Ru3—C1	2.208 (4)	C37'—C38'	1.42 (4)
Ru3—C2	2.164 (4)	C38—C39	1.399 (16)
Ru3—C3	2.170 (5)	C38'—C39'	1.40 (4)
Ru3—C4	2.191 (4)	C39—C40	1.418 (18)
Ru3—C11	2.246 (4)	C39'—C40'	1.41 (3)
Ru3—C14	2.266 (4)	C1—H1	0.9500
Ru3—C36	2.179 (7)	C2—H2	0.9500
Ru3—C37	2.178 (8)	C3—H3	0.9500
Ru3—C38	2.167 (10)	C4—H4	0.9500
Ru3—C39	2.165 (11)	C5—H5	0.9500
Ru3—C40	2.179 (9)	C6—H6	0.9500
Ru3—C36'	2.20 (2)	C7—H7	0.9500
Ru3—C37'	2.12 (2)	C8—H8	0.9500
Ru3—C38'	2.18 (3)	C9—H9	1.0000
Ru3—C39'	2.20 (3)	C10—H10	1.0000
Ru3—C40'	2.24 (2)	C15—H15	1.0000
P1—F1	1.587 (9)	C16—H16	1.0000
P1—F2	1.591 (9)	C17—H17B	0.9900
P1—F3	1.569 (7)	C17—H17A	0.9900
P1—F4	1.580 (6)	C18—H18B	0.9900
P1—F5	1.589 (6)	C18—H18A	0.9900
P1—F6	1.564 (6)	C19—H19B	0.9900
P2—F7	1.594 (3)	C19—H19A	0.9900
P2—F8	1.590 (3)	C21—H21	0.9500
P2—F9	1.585 (3)	C22—H22	0.9500
P2—F10	1.599 (3)	C23—H23	0.9500
P2—F11	1.600 (3)	C24—H24	0.9500
P2—F12	1.582 (3)	C25—H25	0.9500
P3—F13	1.585 (12)	C26—H26	0.9500
P3—F14	1.598 (13)	C27—H27	0.9500
P3—F15	1.565 (11)	C28—H28	0.9500
P3—F16	1.579 (9)	C29—H29	0.9500
P3—F17	1.574 (10)	C30—H30	0.9500
P3—F18	1.563 (9)	C31—H31	0.9500
P1'—F1'	1.59 (3)	C32—H32	0.9500
P1'—F2'	1.60 (3)	C33—H33	0.9500
P1'—F3'	1.57 (2)	C34—H34	0.9500
P1'—F4'	1.57 (2)	C35—H35	0.9500
P1'—F5'	1.58 (2)	C36—H36	0.9500
P1'—F6'	1.55 (2)	C36'—H36'	0.9500
P3'—F13'	1.58 (2)	C37—H37	0.9500
P3'—F14'	1.59 (3)	C37'—H37'	0.9500
P3'—F15'	1.558 (19)	C38—H38	0.9500
P3'—F16'	1.580 (17)	C38'—H38'	0.9500
P3'—F17'	1.572 (19)	C39—H39	0.9500
P3'—F18'	1.561 (17)	C39'—H39'	0.9500
N1—C19	1.449 (5)	C40—H40	0.9500
N1—C17	1.464 (6)	C40'—H40'	0.9500
N1—C18	1.466 (6)	C41—C42	1.479 (10)
O1—C41	1.205 (7)	C41—C43	1.473 (10)
O2—C44	1.188 (12)	C42—H42B	0.9800
O2'—C44'	1.19 (5)	C42—H42C	0.9800
C1—C14	1.411 (6)	C42—H42A	0.9800
C1—C2	1.417 (7)	C43—H43B	0.9800
C2—C3	1.388 (8)	C43—H43A	0.9800
C3—C4	1.404 (6)	C43—H43C	0.9800
C4—C11	1.407 (6)	C44—C45	1.481 (15)
C5—C6	1.419 (6)	C44—C46	1.485 (19)
C5—C12	1.394 (5)	C45—H45B	0.9800
C6—C7	1.403 (7)	C45—H45C	0.9800
C7—C8	1.419 (6)	C45—H45A	0.9800
C8—C13	1.408 (6)	C46—H46B	0.9800
C9—C13	1.521 (5)	C46—H46C	0.9800
C9—C15	1.554 (5)	C46—H46A	0.9800
C9—C14	1.500 (6)	C44'—C45'	1.49 (6)
C10—C16	1.554 (6)	C44'—C46'	1.47 (7)
C10—C11	1.517 (6)	C45'—H45D	0.9800
C10—C12	1.517 (5)	C45'—H45E	0.9800
C11—C14	1.410 (6)	C45'—H45F	0.9800
C12—C13	1.409 (6)	C46'—H46D	0.9800
C15—C18	1.513 (6)	C46'—H46E	0.9800
C15—C16	1.560 (6)	C46'—H46F	0.9700
C16—C17	1.541 (6)		
			
C20—Ru1—C21	36.96 (16)	Ru2—C6—C7	71.6 (2)
C20—Ru1—C22	67.34 (19)	C5—C6—C7	120.3 (4)
C20—Ru1—C23	79.35 (19)	Ru2—C7—C6	71.3 (3)
C20—Ru1—C24	67.16 (18)	Ru2—C7—C8	71.5 (2)
C20—Ru1—C25	36.94 (15)	C6—C7—C8	120.7 (4)
C20—Ru1—C26	116.59 (18)	Ru2—C8—C7	71.0 (3)
C20—Ru1—C27	107.61 (17)	C7—C8—C13	118.5 (4)
C20—Ru1—C28	128.97 (17)	Ru2—C8—C13	71.6 (2)
C20—Ru1—C29	167.01 (17)	C13—C9—C14	107.2 (3)
C20—Ru1—C30	149.30 (18)	C14—C9—C15	108.4 (3)
C21—Ru1—C22	37.85 (19)	C13—C9—C15	105.3 (3)
C21—Ru1—C23	67.85 (19)	C11—C10—C16	108.7 (3)
C21—Ru1—C24	79.64 (18)	C11—C10—C12	106.5 (3)
C21—Ru1—C25	66.74 (16)	C12—C10—C16	105.9 (3)
C21—Ru1—C26	107.39 (19)	Ru3—C11—C4	69.4 (2)
C21—Ru1—C27	123.89 (17)	Ru3—C11—C10	134.0 (3)
C21—Ru1—C28	160.29 (17)	C4—C11—C14	120.5 (4)
C21—Ru1—C29	155.76 (17)	C10—C11—C14	112.9 (4)
C21—Ru1—C30	121.17 (19)	Ru3—C11—C14	72.6 (2)
C22—Ru1—C23	37.7 (2)	C4—C11—C10	126.5 (4)
C22—Ru1—C24	67.3 (2)	Ru2—C12—C13	70.7 (2)
C22—Ru1—C25	79.27 (18)	Ru2—C12—C5	71.1 (2)
C22—Ru1—C26	120.1 (2)	Ru2—C12—C10	134.6 (3)
C22—Ru1—C27	154.36 (18)	C10—C12—C13	112.6 (3)
C22—Ru1—C28	161.75 (19)	C5—C12—C10	126.2 (4)
C22—Ru1—C29	124.77 (19)	C5—C12—C13	121.1 (4)
C22—Ru1—C30	107.5 (2)	Ru2—C13—C9	132.5 (3)
C23—Ru1—C24	36.83 (19)	C8—C13—C12	120.5 (4)
C23—Ru1—C25	66.75 (19)	Ru2—C13—C12	72.4 (2)
C23—Ru1—C26	148.3 (2)	C8—C13—C9	126.1 (4)
C23—Ru1—C27	167.59 (18)	Ru2—C13—C8	71.3 (2)
C23—Ru1—C28	129.58 (19)	C9—C13—C12	113.3 (3)
C23—Ru1—C29	107.38 (19)	Ru3—C14—C9	134.3 (3)
C23—Ru1—C30	115.9 (2)	Ru3—C14—C11	71.0 (2)
C24—Ru1—C25	37.30 (18)	Ru3—C14—C1	69.4 (2)
C24—Ru1—C26	172.4 (2)	C1—C14—C11	120.0 (4)
C24—Ru1—C27	135.73 (18)	C9—C14—C11	113.3 (4)
C24—Ru1—C28	109.01 (18)	C1—C14—C9	126.7 (4)
C24—Ru1—C29	111.35 (18)	C16—C15—C18	105.0 (3)
C24—Ru1—C30	140.9 (2)	C9—C15—C18	114.6 (3)
C25—Ru1—C26	142.34 (18)	C9—C15—C16	109.4 (3)
C25—Ru1—C27	112.44 (17)	C15—C16—C17	103.8 (3)
C25—Ru1—C28	108.99 (17)	C10—C16—C17	115.4 (3)
C25—Ru1—C29	134.88 (16)	C10—C16—C15	109.4 (3)
C25—Ru1—C30	172.04 (19)	N1—C17—C16	105.2 (3)
C26—Ru1—C27	37.72 (19)	N1—C18—C15	105.3 (4)
C26—Ru1—C28	63.39 (19)	N1—C19—C20	115.5 (4)
C26—Ru1—C29	63.39 (19)	Ru1—C20—C25	70.5 (2)
C26—Ru1—C30	37.7 (2)	C19—C20—C21	122.8 (4)
C27—Ru1—C28	38.13 (16)	C19—C20—C25	118.3 (4)
C27—Ru1—C29	63.82 (17)	C21—C20—C25	118.9 (4)
C27—Ru1—C30	63.21 (19)	Ru1—C20—C19	129.6 (3)
C28—Ru1—C29	38.31 (17)	Ru1—C20—C21	69.9 (2)
C28—Ru1—C30	63.33 (19)	Ru1—C21—C20	73.2 (3)
C29—Ru1—C30	37.68 (18)	C20—C21—C22	120.0 (4)
C5—Ru2—C6	37.47 (15)	Ru1—C21—C22	70.1 (3)
C5—Ru2—C7	67.24 (16)	C21—C22—C23	120.2 (5)
C5—Ru2—C8	79.93 (16)	Ru1—C22—C23	72.0 (3)
C5—Ru2—C12	36.48 (14)	Ru1—C22—C21	72.1 (3)
C5—Ru2—C13	66.83 (14)	Ru1—C23—C22	70.3 (3)
C5—Ru2—C31	146.75 (16)	Ru1—C23—C24	72.2 (3)
C5—Ru2—C32	170.45 (16)	C22—C23—C24	120.1 (5)
C5—Ru2—C33	132.48 (16)	Ru1—C24—C23	71.0 (3)
C5—Ru2—C34	109.60 (16)	Ru1—C24—C25	71.5 (3)
C5—Ru2—C35	115.84 (17)	C23—C24—C25	119.6 (5)
C6—Ru2—C7	37.18 (18)	Ru1—C25—C20	72.5 (2)
C6—Ru2—C8	67.61 (17)	Ru1—C25—C24	71.2 (3)
C6—Ru2—C12	66.21 (15)	C20—C25—C24	121.1 (4)
C6—Ru2—C13	79.01 (15)	Ru1—C26—C27	70.7 (3)
C6—Ru2—C31	172.95 (16)	C27—C26—C30	108.2 (5)
C6—Ru2—C32	136.94 (16)	Ru1—C26—C30	71.5 (3)
C6—Ru2—C33	109.47 (16)	Ru1—C27—C26	71.6 (3)
C6—Ru2—C34	110.99 (17)	Ru1—C27—C28	70.5 (3)
C6—Ru2—C35	140.02 (18)	C26—C27—C28	107.9 (4)
C7—Ru2—C8	37.52 (16)	Ru1—C28—C27	71.3 (3)
C7—Ru2—C12	78.23 (15)	C27—C28—C29	108.0 (4)
C7—Ru2—C13	66.74 (15)	Ru1—C28—C29	71.2 (3)
C7—Ru2—C31	143.17 (18)	C28—C29—C30	107.7 (4)
C7—Ru2—C32	112.14 (17)	Ru1—C29—C30	72.0 (3)
C7—Ru2—C33	106.81 (16)	Ru1—C29—C28	70.5 (3)
C7—Ru2—C34	132.05 (16)	Ru1—C30—C26	70.8 (3)
C7—Ru2—C35	169.80 (16)	Ru1—C30—C29	70.3 (3)
C8—Ru2—C12	66.77 (15)	C26—C30—C29	108.2 (4)
C8—Ru2—C13	37.13 (14)	Ru2—C31—C32	70.4 (2)
C8—Ru2—C31	116.44 (17)	C32—C31—C35	107.8 (4)
C8—Ru2—C32	105.46 (17)	Ru2—C31—C35	71.1 (2)
C8—Ru2—C33	125.31 (16)	Ru2—C32—C31	72.0 (3)
C8—Ru2—C34	162.98 (16)	Ru2—C32—C33	71.1 (3)
C8—Ru2—C35	150.54 (17)	C31—C32—C33	109.0 (4)
C12—Ru2—C13	36.92 (15)	Ru2—C33—C32	70.9 (3)
C12—Ru2—C31	120.41 (16)	Ru2—C33—C34	71.6 (3)
C12—Ru2—C32	152.91 (16)	C32—C33—C34	107.5 (4)
C12—Ru2—C33	165.74 (17)	C33—C34—C35	107.7 (4)
C12—Ru2—C34	129.20 (17)	Ru2—C34—C35	71.0 (3)
C12—Ru2—C35	110.18 (16)	Ru2—C34—C33	70.4 (3)
C13—Ru2—C31	107.74 (16)	Ru2—C35—C31	71.2 (3)
C13—Ru2—C32	122.20 (16)	Ru2—C35—C34	71.0 (2)
C13—Ru2—C33	157.34 (17)	C31—C35—C34	108.0 (4)
C13—Ru2—C34	159.41 (16)	Ru3—C36—C40	71.2 (5)
C13—Ru2—C35	123.44 (16)	Ru3—C36—C37	71.0 (4)
C31—Ru2—C32	37.65 (17)	C37—C36—C40	107.7 (8)
C31—Ru2—C33	63.51 (16)	C37'—C36'—C40'	108 (2)
C31—Ru2—C34	63.33 (17)	Ru3—C36'—C40'	73.1 (12)
C31—Ru2—C35	37.67 (18)	Ru3—C36'—C37'	67.5 (14)
C32—Ru2—C33	38.00 (17)	Ru3—C37—C38	70.5 (6)
C32—Ru2—C34	63.24 (18)	Ru3—C37—C36	71.1 (5)
C32—Ru2—C35	63.07 (18)	C36—C37—C38	108.4 (9)
C33—Ru2—C34	38.00 (18)	Ru3—C37'—C36'	74.3 (13)
C33—Ru2—C35	63.65 (17)	C36'—C37'—C38'	108 (2)
C34—Ru2—C35	38.09 (17)	Ru3—C37'—C38'	73.3 (15)
C1—Ru3—C2	37.81 (17)	C37—C38—C39	107.2 (9)
C1—Ru3—C3	67.71 (19)	Ru3—C38—C37	71.4 (5)
C1—Ru3—C4	80.11 (17)	Ru3—C38—C39	71.1 (6)
C1—Ru3—C11	66.53 (16)	Ru3—C38'—C37'	68.3 (14)
C1—Ru3—C14	36.73 (15)	C37'—C38'—C39'	107 (2)
C1—Ru3—C36	139.6 (4)	Ru3—C38'—C39'	72.1 (17)
C1—Ru3—C37	175.7 (2)	Ru3—C39—C38	71.2 (6)
C1—Ru3—C38	139.6 (3)	Ru3—C39—C40	71.5 (6)
C1—Ru3—C39	113.0 (3)	C38—C39—C40	109.0 (9)
C1—Ru3—C40	112.8 (3)	Ru3—C39'—C40'	73.2 (14)
C1—Ru3—C36'	124.5 (9)	Ru3—C39'—C38'	70.5 (15)
C1—Ru3—C37'	161.6 (7)	C38'—C39'—C40'	109.3 (19)
C1—Ru3—C38'	153.1 (7)	Ru3—C40—C39	70.4 (6)
C1—Ru3—C39'	118.9 (8)	Ru3—C40—C36	71.2 (5)
C1—Ru3—C40'	106.7 (6)	C36—C40—C39	107.7 (10)
C2—Ru3—C3	37.4 (2)	Ru3—C40'—C39'	69.9 (15)
C2—Ru3—C4	67.72 (18)	Ru3—C40'—C36'	70.0 (13)
C2—Ru3—C11	78.75 (16)	C36'—C40'—C39'	107 (2)
C2—Ru3—C14	66.58 (16)	C2—C1—H1	121.00
C2—Ru3—C36	168.5 (3)	C14—C1—H1	121.00
C2—Ru3—C37	143.4 (3)	Ru3—C1—H1	128.00
C2—Ru3—C38	111.2 (3)	C1—C2—H2	120.00
C2—Ru3—C39	106.1 (3)	C3—C2—H2	120.00
C2—Ru3—C40	131.0 (4)	Ru3—C2—H2	128.00
C2—Ru3—C36'	151.7 (10)	C4—C3—H3	120.00
C2—Ru3—C37'	159.8 (7)	C2—C3—H3	120.00
C2—Ru3—C38'	122.0 (7)	Ru3—C3—H3	130.00
C2—Ru3—C39'	103.9 (7)	Ru3—C4—H4	127.00
C2—Ru3—C40'	117.0 (6)	C11—C4—H4	120.00
C3—Ru3—C4	37.55 (17)	C3—C4—H4	120.00
C3—Ru3—C11	66.54 (16)	C6—C5—H5	121.00
C3—Ru3—C14	78.46 (16)	C12—C5—H5	121.00
C3—Ru3—C36	151.3 (4)	Ru2—C5—H5	129.00
C3—Ru3—C37	115.5 (3)	Ru2—C6—H6	129.00
C3—Ru3—C38	101.8 (3)	C5—C6—H6	120.00
C3—Ru3—C39	121.5 (3)	C7—C6—H6	120.00
C3—Ru3—C40	159.3 (4)	C6—C7—H7	120.00
C3—Ru3—C36'	167.7 (9)	Ru2—C7—H7	130.00
C3—Ru3—C37'	129.9 (6)	C8—C7—H7	120.00
C3—Ru3—C38'	105.5 (7)	Ru2—C8—H8	129.00
C3—Ru3—C39'	112.2 (6)	C7—C8—H8	121.00
C3—Ru3—C40'	143.7 (7)	C13—C8—H8	121.00
C4—Ru3—C11	36.96 (16)	C15—C9—H9	112.00
C4—Ru3—C14	66.56 (15)	C13—C9—H9	112.00
C4—Ru3—C36	123.7 (4)	C14—C9—H9	112.00
C4—Ru3—C37	104.2 (2)	C12—C10—H10	112.00
C4—Ru3—C38	116.1 (3)	C16—C10—H10	112.00
C4—Ru3—C39	151.1 (3)	C11—C10—H10	112.00
C4—Ru3—C40	161.0 (4)	C9—C15—H15	109.00
C4—Ru3—C36'	139.6 (8)	C18—C15—H15	109.00
C4—Ru3—C37'	110.5 (6)	C16—C15—H15	109.00
C4—Ru3—C38'	111.0 (8)	C15—C16—H16	109.00
C4—Ru3—C39'	138.8 (7)	C10—C16—H16	109.00
C4—Ru3—C40'	173.1 (6)	C17—C16—H16	109.00
C11—Ru3—C14	36.42 (16)	N1—C17—H17B	111.00
C11—Ru3—C36	111.1 (2)	N1—C17—H17A	111.00
C11—Ru3—C37	117.1 (3)	H17A—C17—H17B	109.00
C11—Ru3—C38	147.3 (4)	C16—C17—H17A	111.00
C11—Ru3—C39	171.6 (3)	C16—C17—H17B	111.00
C11—Ru3—C40	133.7 (4)	C15—C18—H18A	111.00
C11—Ru3—C36'	118.0 (5)	N1—C18—H18B	111.00
C11—Ru3—C37'	112.5 (7)	C15—C18—H18B	111.00
C11—Ru3—C38'	136.6 (8)	H18A—C18—H18B	109.00
C11—Ru3—C39'	173.9 (8)	N1—C18—H18A	111.00
C11—Ru3—C40'	146.6 (7)	N1—C19—H19A	108.00
C14—Ru3—C36	117.6 (2)	H19A—C19—H19B	108.00
C14—Ru3—C37	145.3 (3)	N1—C19—H19B	108.00
C14—Ru3—C38	176.0 (3)	C20—C19—H19A	108.00
C14—Ru3—C39	138.9 (3)	C20—C19—H19B	108.00
C14—Ru3—C40	115.0 (3)	Ru1—C21—H21	129.00
C14—Ru3—C36'	112.2 (6)	C20—C21—H21	120.00
C14—Ru3—C37'	132.4 (7)	C22—C21—H21	120.00
C14—Ru3—C38'	170.2 (7)	C23—C22—H22	120.00
C14—Ru3—C39'	149.7 (8)	Ru1—C22—H22	128.00
C14—Ru3—C40'	119.6 (6)	C21—C22—H22	120.00
C36—Ru3—C37	38.0 (4)	C22—C23—H23	120.00
C36—Ru3—C38	63.9 (3)	C24—C23—H23	120.00
C36—Ru3—C39	63.4 (4)	Ru1—C23—H23	130.00
C36—Ru3—C40	37.7 (5)	Ru1—C24—H24	130.00
C37—Ru3—C38	38.1 (4)	C23—C24—H24	120.00
C37—Ru3—C39	63.0 (4)	C25—C24—H24	120.00
C37—Ru3—C40	63.1 (4)	C20—C25—H25	119.00
C38—Ru3—C39	37.7 (4)	C24—C25—H25	119.00
C38—Ru3—C40	63.7 (4)	Ru1—C25—H25	129.00
C39—Ru3—C40	38.1 (5)	C27—C26—H26	126.00
C36'—Ru3—C37'	38.2 (11)	C30—C26—H26	126.00
C36'—Ru3—C38'	63.0 (10)	Ru1—C26—H26	124.00
C36'—Ru3—C39'	62.0 (8)	C28—C27—H27	126.00
C36'—Ru3—C40'	36.9 (10)	C26—C27—H27	126.00
C37'—Ru3—C38'	38.5 (10)	Ru1—C27—H27	124.00
C37'—Ru3—C39'	63.4 (10)	Ru1—C28—H28	123.00
C37'—Ru3—C40'	63.2 (8)	C27—C28—H28	126.00
C38'—Ru3—C39'	37.4 (11)	C29—C28—H28	126.00
C38'—Ru3—C40'	62.4 (9)	C30—C29—H29	126.00
C39'—Ru3—C40'	36.9 (9)	Ru1—C29—H29	123.00
F4—P1—F6	178.2 (5)	C28—C29—H29	126.00
F5—P1—F6	89.3 (4)	C26—C30—H30	126.00
F2—P1—F4	89.8 (4)	Ru1—C30—H30	124.00
F1—P1—F2	179.4 (5)	C29—C30—H30	126.00
F1—P1—F3	90.3 (4)	Ru2—C31—H31	124.00
F1—P1—F4	90.2 (4)	C32—C31—H31	126.00
F1—P1—F5	90.1 (4)	C35—C31—H31	126.00
F1—P1—F6	90.2 (4)	C31—C32—H32	126.00
F2—P1—F3	90.4 (4)	C33—C32—H32	125.00
F3—P1—F4	89.9 (5)	Ru2—C32—H32	123.00
F2—P1—F5	89.3 (4)	Ru2—C33—H33	123.00
F2—P1—F6	89.7 (4)	C32—C33—H33	126.00
F4—P1—F5	89.0 (4)	C34—C33—H33	126.00
F3—P1—F5	178.8 (6)	C35—C34—H34	126.00
F3—P1—F6	91.8 (5)	C33—C34—H34	126.00
F7—P2—F8	179.37 (16)	Ru2—C34—H34	124.00
F11—P2—F12	90.17 (15)	C31—C35—H35	126.00
F8—P2—F11	90.95 (15)	Ru2—C35—H35	123.00
F7—P2—F9	90.38 (15)	C34—C35—H35	126.00
F7—P2—F10	89.95 (15)	C40—C36—H36	126.00
F7—P2—F11	89.28 (15)	Ru3—C36—H36	123.00
F7—P2—F12	90.26 (16)	C37—C36—H36	126.00
F8—P2—F9	89.39 (16)	C40'—C36'—H36'	126.00
F8—P2—F10	89.46 (15)	Ru3—C36'—H36'	125.00
F9—P2—F11	179.03 (16)	C37'—C36'—H36'	126.00
F8—P2—F12	90.33 (16)	C36—C37—H37	126.00
F9—P2—F10	90.06 (16)	C38—C37—H37	126.00
F10—P2—F12	179.17 (16)	Ru3—C37—H37	125.00
F9—P2—F12	90.74 (16)	Ru3—C37'—H37'	118.00
F10—P2—F11	89.03 (15)	C36'—C37'—H37'	126.00
F13—P3—F14	178.1 (7)	C38'—C37'—H37'	126.00
F17—P3—F18	87.2 (5)	Ru3—C38—H38	123.00
F14—P3—F16	90.1 (6)	C39—C38—H38	126.00
F14—P3—F17	89.0 (6)	C37—C38—H38	126.00
F13—P3—F15	93.0 (6)	Ru3—C38'—H38'	125.00
F13—P3—F16	88.4 (6)	C37'—C38'—H38'	126.00
F13—P3—F17	89.8 (6)	C39'—C38'—H38'	126.00
F13—P3—F18	91.3 (6)	C40—C39—H39	126.00
F14—P3—F15	88.2 (6)	C38—C39—H39	126.00
F16—P3—F18	176.7 (6)	Ru3—C39—H39	123.00
F15—P3—F17	177.1 (6)	C40'—C39'—H39'	125.00
F14—P3—F18	90.0 (6)	C38'—C39'—H39'	125.00
F15—P3—F16	91.3 (6)	Ru3—C39'—H39'	123.00
F15—P3—F18	92.0 (5)	C39—C40—H40	126.00
F16—P3—F17	89.5 (5)	C36—C40—H40	126.00
F1'—P1'—F4'	89.2 (15)	Ru3—C40—H40	124.00
F1'—P1'—F3'	90.3 (14)	C36'—C40'—H40'	126.00
F1'—P1'—F2'	177.8 (17)	C39'—C40'—H40'	127.00
F3'—P1'—F4'	87.2 (13)	Ru3—C40'—H40'	125.00
F1'—P1'—F5'	92.1 (15)	O1—C41—C42	121.2 (6)
F1'—P1'—F6'	93.5 (15)	O1—C41—C43	121.8 (6)
F2'—P1'—F3'	89.5 (15)	C42—C41—C43	117.1 (6)
F2'—P1'—F4'	88.7 (15)	C41—C42—H42A	109.00
F2'—P1'—F5'	88.1 (14)	C41—C42—H42B	109.00
F2'—P1'—F6'	88.7 (16)	C41—C42—H42C	109.00
F4'—P1'—F5'	91.0 (14)	H42A—C42—H42B	109.00
F3'—P1'—F5'	177.0 (16)	H42A—C42—H42C	109.00
F3'—P1'—F6'	89.1 (14)	H42B—C42—H42C	110.00
F4'—P1'—F6'	175.5 (17)	C41—C43—H43A	109.00
F5'—P1'—F6'	92.5 (13)	C41—C43—H43B	109.00
F13'—P3'—F16'	93.3 (11)	C41—C43—H43C	109.00
F13'—P3'—F14'	177.8 (14)	H43A—C43—H43B	109.00
F13'—P3'—F15'	89.0 (11)	H43A—C43—H43C	110.00
F14'—P3'—F17'	88.5 (12)	H43B—C43—H43C	110.00
F14'—P3'—F18'	89.6 (12)	O2—C44—C45	120.0 (12)
F15'—P3'—F16'	90.4 (10)	O2—C44—C46	122.8 (10)
F13'—P3'—F17'	91.3 (11)	C45—C44—C46	117.1 (9)
F13'—P3'—F18'	88.2 (11)	C44—C45—H45A	109.00
F14'—P3'—F15'	91.2 (11)	C44—C45—H45B	109.00
F14'—P3'—F16'	88.9 (11)	C44—C45—H45C	109.00
F17'—P3'—F18'	88.2 (11)	H45A—C45—H45B	110.00
F15'—P3'—F18'	92.3 (11)	H45A—C45—H45C	109.00
F16'—P3'—F17'	89.1 (10)	H45B—C45—H45C	110.00
F15'—P3'—F17'	179.4 (12)	C44—C46—H46A	109.00
F16'—P3'—F18'	177.0 (13)	C44—C46—H46B	109.00
C17—N1—C19	114.1 (3)	C44—C46—H46C	110.00
C18—N1—C19	110.1 (3)	H46A—C46—H46B	109.00
C17—N1—C18	104.8 (3)	H46A—C46—H46C	110.00
Ru3—C1—C2	69.4 (3)	H46B—C46—H46C	109.00
Ru3—C1—C14	73.9 (2)	O2'—C44'—C45'	118 (5)
C2—C1—C14	118.7 (4)	O2'—C44'—C46'	122 (4)
Ru3—C2—C3	71.6 (3)	C45'—C44'—C46'	119 (4)
C1—C2—C3	120.8 (4)	C44'—C45'—H45D	109.00
Ru3—C2—C1	72.8 (2)	C44'—C45'—H45E	109.00
Ru3—C3—C2	71.1 (3)	C44'—C45'—H45F	110.00
Ru3—C3—C4	72.0 (3)	H45D—C45'—H45E	109.00
C2—C3—C4	120.7 (4)	H45D—C45'—H45F	109.00
Ru3—C4—C3	70.4 (3)	H45E—C45'—H45F	110.00
C3—C4—C11	119.1 (5)	C44'—C46'—H46D	109.00
Ru3—C4—C11	73.7 (3)	C44'—C46'—H46E	109.00
Ru2—C5—C12	72.4 (2)	C44'—C46'—H46F	110.00
C6—C5—C12	118.9 (4)	H46D—C46'—H46E	109.00
Ru2—C5—C6	70.6 (2)	H46D—C46'—H46F	110.00
Ru2—C6—C5	72.0 (2)	H46E—C46'—H46F	110.00
			
C21—Ru1—C20—C19	−116.4 (5)	C13—Ru2—C35—C34	−167.4 (3)
C21—Ru1—C20—C25	132.4 (4)	C31—Ru2—C35—C34	117.5 (4)
C22—Ru1—C20—C19	−146.2 (4)	C32—Ru2—C35—C31	−37.3 (3)
C22—Ru1—C20—C21	−29.7 (3)	C32—Ru2—C35—C34	80.2 (3)
C22—Ru1—C20—C25	102.7 (3)	C33—Ru2—C35—C31	−80.0 (3)
C23—Ru1—C20—C19	176.4 (4)	C33—Ru2—C35—C34	37.5 (3)
C23—Ru1—C20—C21	−67.2 (3)	C34—Ru2—C35—C31	−117.5 (4)
C23—Ru1—C20—C25	65.3 (3)	C2—Ru3—C1—C14	−130.0 (4)
C24—Ru1—C20—C19	140.0 (4)	C3—Ru3—C1—C2	29.2 (3)
C24—Ru1—C20—C21	−103.6 (3)	C3—Ru3—C1—C14	−100.8 (3)
C24—Ru1—C20—C25	28.8 (3)	C4—Ru3—C1—C2	66.2 (3)
C25—Ru1—C20—C19	111.1 (5)	C4—Ru3—C1—C14	−63.8 (3)
C25—Ru1—C20—C21	−132.4 (4)	C11—Ru3—C1—C2	102.3 (3)
C26—Ru1—C20—C19	−32.7 (4)	C11—Ru3—C1—C14	−27.8 (2)
C26—Ru1—C20—C21	83.7 (3)	C14—Ru3—C1—C2	130.0 (4)
C26—Ru1—C20—C25	−143.9 (3)	C36—Ru3—C1—C2	−162.2 (5)
C27—Ru1—C20—C19	7.0 (4)	C36—Ru3—C1—C14	67.8 (5)
C27—Ru1—C20—C21	123.4 (3)	C38—Ru3—C1—C2	−52.7 (6)
C27—Ru1—C20—C25	−104.1 (3)	C38—Ru3—C1—C14	177.3 (5)
C28—Ru1—C20—C19	43.4 (4)	C39—Ru3—C1—C2	−86.8 (4)
C28—Ru1—C20—C21	159.9 (3)	C39—Ru3—C1—C14	143.2 (4)
C28—Ru1—C20—C25	−67.7 (3)	C40—Ru3—C1—C2	−128.3 (5)
C30—Ru1—C20—C19	−60.0 (6)	C40—Ru3—C1—C14	101.7 (5)
C30—Ru1—C20—C21	56.5 (5)	C1—Ru3—C2—C3	131.9 (4)
C30—Ru1—C20—C25	−171.1 (4)	C3—Ru3—C2—C1	−131.9 (4)
C20—Ru1—C21—C22	−131.8 (4)	C4—Ru3—C2—C1	−103.0 (3)
C22—Ru1—C21—C20	131.8 (4)	C4—Ru3—C2—C3	28.9 (2)
C23—Ru1—C21—C20	102.1 (3)	C11—Ru3—C2—C1	−66.1 (3)
C23—Ru1—C21—C22	−29.7 (3)	C11—Ru3—C2—C3	65.9 (3)
C24—Ru1—C21—C20	65.6 (3)	C14—Ru3—C2—C1	−29.9 (2)
C24—Ru1—C21—C22	−66.2 (3)	C14—Ru3—C2—C3	102.0 (3)
C25—Ru1—C21—C20	28.9 (3)	C37—Ru3—C2—C1	173.1 (4)
C25—Ru1—C21—C22	−102.9 (3)	C37—Ru3—C2—C3	−54.9 (5)
C26—Ru1—C21—C20	−111.3 (3)	C38—Ru3—C2—C1	146.5 (4)
C26—Ru1—C21—C22	116.9 (3)	C38—Ru3—C2—C3	−81.6 (4)
C27—Ru1—C21—C20	−73.4 (3)	C39—Ru3—C2—C1	107.0 (4)
C27—Ru1—C21—C22	154.9 (3)	C39—Ru3—C2—C3	−121.1 (4)
C29—Ru1—C21—C20	−174.7 (4)	C40—Ru3—C2—C1	73.5 (5)
C29—Ru1—C21—C22	53.5 (5)	C40—Ru3—C2—C3	−154.5 (5)
C30—Ru1—C21—C20	−150.2 (3)	C1—Ru3—C3—C2	−29.5 (2)
C30—Ru1—C21—C22	78.1 (3)	C1—Ru3—C3—C4	103.2 (3)
C20—Ru1—C22—C21	29.1 (3)	C2—Ru3—C3—C4	132.8 (4)
C20—Ru1—C22—C23	−102.3 (3)	C4—Ru3—C3—C2	−132.8 (4)
C21—Ru1—C22—C23	−131.4 (4)	C11—Ru3—C3—C2	−102.6 (3)
C23—Ru1—C22—C21	131.4 (4)	C11—Ru3—C3—C4	30.2 (3)
C24—Ru1—C22—C21	102.8 (3)	C14—Ru3—C3—C2	−66.4 (3)
C24—Ru1—C22—C23	−28.6 (3)	C14—Ru3—C3—C4	66.4 (3)
C25—Ru1—C22—C21	65.7 (3)	C36—Ru3—C3—C2	165.9 (5)
C25—Ru1—C22—C23	−65.7 (3)	C36—Ru3—C3—C4	−61.3 (5)
C26—Ru1—C22—C21	−79.5 (3)	C37—Ru3—C3—C2	147.3 (4)
C26—Ru1—C22—C23	149.1 (3)	C37—Ru3—C3—C4	−80.0 (4)
C27—Ru1—C22—C21	−54.5 (5)	C38—Ru3—C3—C2	109.6 (4)
C27—Ru1—C22—C23	174.1 (4)	C38—Ru3—C3—C4	−117.7 (4)
C29—Ru1—C22—C21	−156.3 (3)	C39—Ru3—C3—C2	74.6 (4)
C29—Ru1—C22—C23	72.3 (4)	C39—Ru3—C3—C4	−152.6 (4)
C30—Ru1—C22—C21	−118.6 (3)	C40—Ru3—C3—C2	66.2 (10)
C30—Ru1—C22—C23	110.0 (3)	C40—Ru3—C3—C4	−161.0 (9)
C20—Ru1—C23—C22	66.5 (3)	C1—Ru3—C4—C3	−66.1 (3)
C20—Ru1—C23—C24	−65.9 (3)	C1—Ru3—C4—C11	63.9 (3)
C21—Ru1—C23—C22	29.8 (3)	C2—Ru3—C4—C3	−28.8 (3)
C21—Ru1—C23—C24	−102.7 (3)	C2—Ru3—C4—C11	101.2 (3)
C22—Ru1—C23—C24	−132.5 (5)	C3—Ru3—C4—C11	130.0 (4)
C24—Ru1—C23—C22	132.5 (5)	C11—Ru3—C4—C3	−130.0 (4)
C25—Ru1—C23—C22	103.0 (3)	C14—Ru3—C4—C3	−101.9 (3)
C25—Ru1—C23—C24	−29.5 (3)	C14—Ru3—C4—C11	28.1 (2)
C26—Ru1—C23—C22	−57.6 (5)	C36—Ru3—C4—C3	149.6 (4)
C26—Ru1—C23—C24	169.9 (4)	C36—Ru3—C4—C11	−80.5 (4)
C28—Ru1—C23—C22	−161.0 (3)	C37—Ru3—C4—C3	113.5 (4)
C28—Ru1—C23—C24	66.5 (4)	C37—Ru3—C4—C11	−116.5 (4)
C29—Ru1—C23—C22	−124.9 (3)	C38—Ru3—C4—C3	74.7 (4)
C29—Ru1—C23—C24	102.6 (3)	C38—Ru3—C4—C11	−155.3 (4)
C30—Ru1—C23—C22	−85.3 (3)	C39—Ru3—C4—C3	54.2 (7)
C30—Ru1—C23—C24	142.3 (3)	C39—Ru3—C4—C11	−175.8 (6)
C20—Ru1—C24—C23	103.2 (3)	C1—Ru3—C11—C4	−105.4 (3)
C20—Ru1—C24—C25	−28.6 (3)	C1—Ru3—C11—C10	133.2 (5)
C21—Ru1—C24—C23	66.7 (3)	C1—Ru3—C11—C14	28.0 (2)
C21—Ru1—C24—C25	−65.0 (3)	C2—Ru3—C11—C4	−67.8 (3)
C22—Ru1—C24—C23	29.3 (3)	C2—Ru3—C11—C10	170.9 (5)
C22—Ru1—C24—C25	−102.5 (3)	C2—Ru3—C11—C14	65.6 (3)
C23—Ru1—C24—C25	−131.8 (5)	C3—Ru3—C11—C4	−30.6 (3)
C25—Ru1—C24—C23	131.8 (5)	C3—Ru3—C11—C10	−152.0 (5)
C27—Ru1—C24—C23	−164.6 (3)	C3—Ru3—C11—C14	102.8 (3)
C27—Ru1—C24—C25	63.6 (4)	C4—Ru3—C11—C10	−121.4 (6)
C28—Ru1—C24—C23	−131.6 (3)	C4—Ru3—C11—C14	133.4 (4)
C28—Ru1—C24—C25	96.6 (3)	C14—Ru3—C11—C4	−133.4 (4)
C29—Ru1—C24—C23	−90.8 (3)	C14—Ru3—C11—C10	105.3 (5)
C29—Ru1—C24—C25	137.5 (3)	C36—Ru3—C11—C4	118.4 (5)
C30—Ru1—C24—C23	−60.8 (5)	C36—Ru3—C11—C10	−3.0 (6)
C30—Ru1—C24—C25	167.4 (3)	C36—Ru3—C11—C14	−108.2 (4)
C20—Ru1—C25—C24	132.8 (4)	C37—Ru3—C11—C4	77.2 (4)
C21—Ru1—C25—C20	−28.9 (3)	C37—Ru3—C11—C10	−44.2 (6)
C21—Ru1—C25—C24	103.9 (3)	C37—Ru3—C11—C14	−149.5 (4)
C22—Ru1—C25—C20	−66.4 (3)	C38—Ru3—C11—C4	44.1 (6)
C22—Ru1—C25—C24	66.4 (3)	C38—Ru3—C11—C10	−77.3 (7)
C23—Ru1—C25—C20	−103.7 (3)	C38—Ru3—C11—C14	177.4 (5)
C23—Ru1—C25—C24	29.1 (3)	C40—Ru3—C11—C4	154.9 (5)
C24—Ru1—C25—C20	−132.8 (4)	C40—Ru3—C11—C10	33.5 (7)
C26—Ru1—C25—C20	59.7 (4)	C40—Ru3—C11—C14	−71.8 (5)
C26—Ru1—C25—C24	−167.5 (3)	C1—Ru3—C14—C9	121.6 (5)
C27—Ru1—C25—C20	89.8 (3)	C1—Ru3—C14—C11	−134.0 (4)
C27—Ru1—C25—C24	−137.4 (3)	C2—Ru3—C14—C1	30.8 (3)
C28—Ru1—C25—C20	130.5 (3)	C2—Ru3—C14—C9	152.4 (4)
C28—Ru1—C25—C24	−96.7 (3)	C2—Ru3—C14—C11	−103.2 (3)
C29—Ru1—C25—C20	164.5 (3)	C3—Ru3—C14—C1	68.1 (3)
C29—Ru1—C25—C24	−62.7 (4)	C3—Ru3—C14—C9	−170.3 (4)
C20—Ru1—C26—C27	84.6 (3)	C3—Ru3—C14—C11	−66.0 (3)
C20—Ru1—C26—C30	−157.6 (3)	C4—Ru3—C14—C1	105.6 (3)
C21—Ru1—C26—C27	123.4 (3)	C4—Ru3—C14—C9	−132.8 (4)
C21—Ru1—C26—C30	−118.8 (3)	C4—Ru3—C14—C11	−28.5 (2)
C22—Ru1—C26—C27	162.6 (3)	C11—Ru3—C14—C1	134.0 (4)
C22—Ru1—C26—C30	−79.6 (4)	C11—Ru3—C14—C9	−104.4 (5)
C23—Ru1—C26—C27	−160.8 (3)	C36—Ru3—C14—C1	−137.3 (5)
C23—Ru1—C26—C30	−43.0 (5)	C36—Ru3—C14—C9	−15.7 (6)
C25—Ru1—C26—C27	49.1 (4)	C36—Ru3—C14—C11	88.7 (5)
C25—Ru1—C26—C30	167.0 (3)	C37—Ru3—C14—C1	−173.5 (4)
C27—Ru1—C26—C30	117.8 (4)	C37—Ru3—C14—C9	−51.8 (6)
C28—Ru1—C26—C27	−37.8 (3)	C37—Ru3—C14—C11	52.5 (5)
C28—Ru1—C26—C30	80.0 (3)	C39—Ru3—C14—C1	−57.1 (5)
C29—Ru1—C26—C27	−80.9 (3)	C39—Ru3—C14—C9	64.5 (6)
C29—Ru1—C26—C30	37.0 (3)	C39—Ru3—C14—C11	168.9 (5)
C30—Ru1—C26—C27	−117.8 (4)	C40—Ru3—C14—C1	−95.2 (4)
C20—Ru1—C27—C26	−110.9 (3)	C40—Ru3—C14—C9	26.4 (6)
C20—Ru1—C27—C28	131.6 (3)	C40—Ru3—C14—C11	130.8 (4)
C21—Ru1—C27—C26	−73.7 (3)	C1—Ru3—C36—C37	174.4 (5)
C21—Ru1—C27—C28	168.8 (2)	C1—Ru3—C36—C40	57.2 (7)
C22—Ru1—C27—C26	−36.8 (5)	C3—Ru3—C36—C37	−28.0 (8)
C22—Ru1—C27—C28	−154.2 (4)	C3—Ru3—C36—C40	−145.2 (7)
C24—Ru1—C27—C26	174.0 (3)	C4—Ru3—C36—C37	−68.0 (6)
C24—Ru1—C27—C28	56.6 (4)	C4—Ru3—C36—C40	174.9 (6)
C25—Ru1—C27—C26	−150.0 (3)	C11—Ru3—C36—C37	−107.4 (5)
C25—Ru1—C27—C28	92.5 (3)	C11—Ru3—C36—C40	135.4 (6)
C26—Ru1—C27—C28	−117.5 (4)	C14—Ru3—C36—C37	−147.0 (5)
C28—Ru1—C27—C26	117.5 (4)	C14—Ru3—C36—C40	95.9 (6)
C29—Ru1—C27—C26	79.6 (3)	C37—Ru3—C36—C40	−117.2 (8)
C29—Ru1—C27—C28	−37.8 (3)	C38—Ru3—C36—C37	37.2 (6)
C30—Ru1—C27—C26	37.3 (3)	C38—Ru3—C36—C40	−80.0 (7)
C30—Ru1—C27—C28	−80.1 (3)	C39—Ru3—C36—C37	79.5 (6)
C20—Ru1—C28—C27	−66.4 (3)	C39—Ru3—C36—C40	−37.7 (6)
C20—Ru1—C28—C29	176.2 (2)	C40—Ru3—C36—C37	117.2 (8)
C23—Ru1—C28—C27	−177.5 (3)	C2—Ru3—C37—C36	−161.1 (5)
C23—Ru1—C28—C29	65.1 (3)	C2—Ru3—C37—C38	−42.6 (8)
C24—Ru1—C28—C27	−142.0 (3)	C3—Ru3—C37—C36	165.6 (5)
C24—Ru1—C28—C29	100.7 (3)	C3—Ru3—C37—C38	−76.0 (6)
C25—Ru1—C28—C27	−102.4 (3)	C4—Ru3—C37—C36	127.3 (5)
C25—Ru1—C28—C29	140.2 (3)	C4—Ru3—C37—C38	−114.3 (6)
C26—Ru1—C28—C27	37.4 (3)	C11—Ru3—C37—C36	90.1 (6)
C26—Ru1—C28—C29	−80.0 (3)	C11—Ru3—C37—C38	−151.5 (6)
C27—Ru1—C28—C29	−117.4 (4)	C14—Ru3—C37—C36	58.1 (7)
C29—Ru1—C28—C27	117.4 (4)	C14—Ru3—C37—C38	176.5 (5)
C30—Ru1—C28—C27	79.8 (3)	C36—Ru3—C37—C38	118.4 (8)
C30—Ru1—C28—C29	−37.6 (3)	C38—Ru3—C37—C36	−118.4 (8)
C21—Ru1—C29—C28	152.6 (4)	C39—Ru3—C37—C36	−80.5 (6)
C21—Ru1—C29—C30	35.6 (6)	C39—Ru3—C37—C38	37.9 (6)
C22—Ru1—C29—C28	−170.5 (3)	C40—Ru3—C37—C36	−37.6 (6)
C22—Ru1—C29—C30	72.5 (4)	C40—Ru3—C37—C38	80.9 (7)
C23—Ru1—C29—C28	−132.9 (3)	C1—Ru3—C38—C37	−174.1 (4)
C23—Ru1—C29—C30	110.1 (3)	C1—Ru3—C38—C39	−57.7 (8)
C24—Ru1—C29—C28	−94.0 (3)	C2—Ru3—C38—C37	154.4 (5)
C24—Ru1—C29—C30	149.0 (3)	C2—Ru3—C38—C39	−89.2 (6)
C25—Ru1—C29—C28	−58.7 (4)	C3—Ru3—C38—C37	116.5 (5)
C25—Ru1—C29—C30	−175.7 (3)	C3—Ru3—C38—C39	−127.0 (6)
C26—Ru1—C29—C28	80.0 (3)	C4—Ru3—C38—C37	79.6 (6)
C26—Ru1—C29—C30	−37.0 (3)	C4—Ru3—C38—C39	−163.9 (5)
C27—Ru1—C29—C28	37.7 (3)	C11—Ru3—C38—C37	51.9 (8)
C27—Ru1—C29—C30	−79.3 (3)	C11—Ru3—C38—C39	168.4 (5)
C28—Ru1—C29—C30	−117.0 (4)	C36—Ru3—C38—C37	−37.0 (6)
C30—Ru1—C29—C28	117.0 (4)	C36—Ru3—C38—C39	79.4 (7)
C20—Ru1—C30—C26	42.0 (5)	C37—Ru3—C38—C39	116.5 (8)
C20—Ru1—C30—C29	160.3 (3)	C39—Ru3—C38—C37	−116.5 (8)
C21—Ru1—C30—C26	77.8 (4)	C40—Ru3—C38—C37	−79.2 (6)
C21—Ru1—C30—C29	−163.8 (3)	C40—Ru3—C38—C39	37.3 (7)
C22—Ru1—C30—C26	116.8 (3)	C1—Ru3—C39—C38	143.5 (5)
C22—Ru1—C30—C29	−124.8 (3)	C1—Ru3—C39—C40	−98.1 (6)
C23—Ru1—C30—C26	156.5 (3)	C2—Ru3—C39—C38	103.9 (6)
C23—Ru1—C30—C29	−85.1 (3)	C2—Ru3—C39—C40	−137.7 (6)
C24—Ru1—C30—C26	−167.9 (3)	C3—Ru3—C39—C38	66.4 (6)
C24—Ru1—C30—C29	−49.5 (5)	C3—Ru3—C39—C40	−175.2 (5)
C26—Ru1—C30—C29	118.4 (5)	C4—Ru3—C39—C38	31.0 (10)
C27—Ru1—C30—C26	−37.3 (3)	C4—Ru3—C39—C40	149.4 (6)
C27—Ru1—C30—C29	81.1 (3)	C14—Ru3—C39—C38	176.6 (4)
C28—Ru1—C30—C26	−80.2 (3)	C14—Ru3—C39—C40	−65.0 (8)
C28—Ru1—C30—C29	38.2 (3)	C36—Ru3—C39—C38	−81.1 (7)
C29—Ru1—C30—C26	−118.4 (5)	C36—Ru3—C39—C40	37.3 (7)
C6—Ru2—C5—C12	130.5 (4)	C37—Ru3—C39—C38	−38.4 (6)
C7—Ru2—C5—C6	−29.2 (2)	C37—Ru3—C39—C40	80.1 (7)
C7—Ru2—C5—C12	101.3 (3)	C38—Ru3—C39—C40	118.4 (9)
C8—Ru2—C5—C6	−66.2 (3)	C40—Ru3—C39—C38	−118.4 (9)
C8—Ru2—C5—C12	64.3 (2)	C1—Ru3—C40—C36	−143.8 (5)
C12—Ru2—C5—C6	−130.5 (4)	C1—Ru3—C40—C39	98.7 (6)
C13—Ru2—C5—C6	−102.6 (3)	C2—Ru3—C40—C36	176.6 (4)
C13—Ru2—C5—C12	27.9 (2)	C2—Ru3—C40—C39	59.1 (7)
C31—Ru2—C5—C6	170.2 (3)	C3—Ru3—C40—C36	129.2 (9)
C31—Ru2—C5—C12	−59.3 (4)	C3—Ru3—C40—C39	11.7 (13)
C33—Ru2—C5—C6	63.2 (3)	C11—Ru3—C40—C36	−65.0 (7)
C33—Ru2—C5—C12	−166.4 (2)	C11—Ru3—C40—C39	177.4 (5)
C34—Ru2—C5—C6	99.2 (3)	C14—Ru3—C40—C36	−103.5 (6)
C34—Ru2—C5—C12	−130.3 (2)	C14—Ru3—C40—C39	138.9 (5)
C35—Ru2—C5—C6	140.1 (3)	C36—Ru3—C40—C39	−117.6 (9)
C35—Ru2—C5—C12	−89.4 (3)	C37—Ru3—C40—C36	37.8 (6)
C5—Ru2—C6—C7	−131.9 (4)	C37—Ru3—C40—C39	−79.7 (7)
C7—Ru2—C6—C5	131.9 (4)	C38—Ru3—C40—C36	80.7 (6)
C8—Ru2—C6—C5	103.1 (3)	C38—Ru3—C40—C39	−36.9 (6)
C8—Ru2—C6—C7	−28.8 (2)	C39—Ru3—C40—C36	117.6 (9)
C12—Ru2—C6—C5	29.6 (2)	C18—N1—C17—C16	38.7 (4)
C12—Ru2—C6—C7	−102.3 (3)	C19—N1—C17—C16	159.1 (3)
C13—Ru2—C6—C5	66.1 (2)	C17—N1—C18—C15	−39.4 (4)
C13—Ru2—C6—C7	−65.8 (2)	C19—N1—C18—C15	−162.5 (3)
C32—Ru2—C6—C5	−168.0 (3)	C17—N1—C19—C20	73.0 (5)
C32—Ru2—C6—C7	60.1 (3)	C18—N1—C19—C20	−169.6 (4)
C33—Ru2—C6—C5	−135.7 (2)	Ru3—C1—C2—C3	−55.2 (4)
C33—Ru2—C6—C7	92.4 (3)	C14—C1—C2—Ru3	57.0 (3)
C34—Ru2—C6—C5	−95.2 (3)	C14—C1—C2—C3	1.8 (6)
C34—Ru2—C6—C7	132.9 (2)	Ru3—C1—C14—C9	−130.6 (4)
C35—Ru2—C6—C5	−63.9 (3)	Ru3—C1—C14—C11	51.8 (3)
C35—Ru2—C6—C7	164.2 (3)	C2—C1—C14—Ru3	−54.8 (3)
C5—Ru2—C7—C6	29.4 (2)	C2—C1—C14—C9	174.6 (4)
C5—Ru2—C7—C8	−103.6 (3)	C2—C1—C14—C11	−3.1 (6)
C6—Ru2—C7—C8	−133.0 (4)	Ru3—C2—C3—C4	−54.3 (4)
C8—Ru2—C7—C6	133.0 (4)	C1—C2—C3—Ru3	55.8 (4)
C12—Ru2—C7—C6	66.0 (2)	C1—C2—C3—C4	1.5 (7)
C12—Ru2—C7—C8	−67.0 (3)	Ru3—C3—C4—C11	−57.3 (3)
C13—Ru2—C7—C6	102.9 (3)	C2—C3—C4—Ru3	53.9 (4)
C13—Ru2—C7—C8	−30.1 (2)	C2—C3—C4—C11	−3.4 (6)
C31—Ru2—C7—C6	−168.3 (3)	Ru3—C4—C11—C10	130.1 (4)
C31—Ru2—C7—C8	58.7 (4)	Ru3—C4—C11—C14	−53.6 (3)
C32—Ru2—C7—C6	−140.3 (2)	C3—C4—C11—Ru3	55.7 (3)
C32—Ru2—C7—C8	86.7 (3)	C3—C4—C11—C10	−174.2 (4)
C33—Ru2—C7—C6	−100.3 (3)	C3—C4—C11—C14	2.1 (6)
C33—Ru2—C7—C8	126.7 (3)	Ru2—C5—C6—C7	54.9 (4)
C34—Ru2—C7—C6	−67.0 (3)	C12—C5—C6—Ru2	−55.9 (3)
C34—Ru2—C7—C8	160.0 (3)	C12—C5—C6—C7	−1.1 (6)
C5—Ru2—C8—C7	65.6 (3)	Ru2—C5—C12—C10	132.0 (4)
C5—Ru2—C8—C13	−64.7 (3)	Ru2—C5—C12—C13	−52.1 (4)
C6—Ru2—C8—C7	28.6 (2)	C6—C5—C12—Ru2	55.0 (3)
C6—Ru2—C8—C13	−101.7 (3)	C6—C5—C12—C10	−173.0 (4)
C7—Ru2—C8—C13	−130.2 (4)	C6—C5—C12—C13	2.9 (6)
C12—Ru2—C8—C7	101.2 (3)	Ru2—C6—C7—C8	53.8 (4)
C12—Ru2—C8—C13	−29.0 (2)	C5—C6—C7—Ru2	−55.1 (4)
C13—Ru2—C8—C7	130.2 (4)	C5—C6—C7—C8	−1.3 (6)
C31—Ru2—C8—C7	−145.1 (3)	Ru2—C7—C8—C13	55.5 (3)
C31—Ru2—C8—C13	84.7 (3)	C6—C7—C8—Ru2	−53.7 (4)
C32—Ru2—C8—C7	−106.4 (3)	C6—C7—C8—C13	1.8 (6)
C32—Ru2—C8—C13	123.4 (3)	Ru2—C8—C13—C9	−129.5 (4)
C33—Ru2—C8—C7	−70.1 (3)	Ru2—C8—C13—C12	55.2 (4)
C33—Ru2—C8—C13	159.7 (2)	C7—C8—C13—Ru2	−55.2 (3)
C35—Ru2—C8—C7	−168.6 (3)	C7—C8—C13—C9	175.3 (4)
C35—Ru2—C8—C13	61.2 (4)	C7—C8—C13—C12	0.0 (6)
C5—Ru2—C12—C10	−122.6 (5)	C14—C9—C13—Ru2	33.1 (5)
C5—Ru2—C12—C13	134.3 (4)	C14—C9—C13—C8	130.6 (4)
C6—Ru2—C12—C5	−30.4 (2)	C14—C9—C13—C12	−53.8 (4)
C6—Ru2—C12—C10	−153.0 (5)	C15—C9—C13—Ru2	148.5 (3)
C6—Ru2—C12—C13	103.9 (3)	C15—C9—C13—C8	−114.1 (4)
C7—Ru2—C12—C5	−67.5 (3)	C15—C9—C13—C12	61.6 (4)
C7—Ru2—C12—C10	169.9 (4)	C13—C9—C14—Ru3	140.4 (3)
C7—Ru2—C12—C13	66.8 (3)	C13—C9—C14—C1	−123.1 (4)
C8—Ru2—C12—C5	−105.1 (3)	C13—C9—C14—C11	54.7 (4)
C8—Ru2—C12—C10	132.3 (4)	C15—C9—C14—Ru3	27.1 (5)
C8—Ru2—C12—C13	29.2 (2)	C15—C9—C14—C1	123.6 (4)
C13—Ru2—C12—C5	−134.3 (4)	C15—C9—C14—C11	−58.6 (4)
C13—Ru2—C12—C10	103.1 (5)	C13—C9—C15—C16	−57.4 (4)
C31—Ru2—C12—C5	146.9 (3)	C13—C9—C15—C18	60.3 (4)
C31—Ru2—C12—C10	24.2 (5)	C14—C9—C15—C16	57.2 (4)
C31—Ru2—C12—C13	−78.9 (3)	C14—C9—C15—C18	174.8 (3)
C32—Ru2—C12—C5	176.6 (3)	C12—C10—C11—Ru3	−142.9 (4)
C32—Ru2—C12—C10	53.9 (6)	C12—C10—C11—C4	121.0 (4)
C32—Ru2—C12—C13	−49.2 (5)	C12—C10—C11—C14	−55.5 (4)
C34—Ru2—C12—C5	68.0 (3)	C16—C10—C11—Ru3	−29.2 (6)
C34—Ru2—C12—C10	−54.6 (5)	C16—C10—C11—C4	−125.3 (4)
C34—Ru2—C12—C13	−157.8 (2)	C16—C10—C11—C14	58.2 (4)
C35—Ru2—C12—C5	106.5 (3)	C11—C10—C12—Ru2	−28.5 (5)
C35—Ru2—C12—C10	−16.1 (5)	C11—C10—C12—C5	−127.6 (4)
C35—Ru2—C12—C13	−119.2 (3)	C11—C10—C12—C13	56.2 (5)
C5—Ru2—C13—C8	104.5 (3)	C16—C10—C12—Ru2	−144.1 (3)
C5—Ru2—C13—C9	−133.3 (4)	C16—C10—C12—C5	116.8 (5)
C5—Ru2—C13—C12	−27.6 (2)	C16—C10—C12—C13	−59.4 (4)
C6—Ru2—C13—C8	67.3 (3)	C11—C10—C16—C15	−55.2 (4)
C6—Ru2—C13—C9	−170.5 (4)	C11—C10—C16—C17	−171.7 (4)
C6—Ru2—C13—C12	−64.8 (3)	C12—C10—C16—C15	59.0 (4)
C7—Ru2—C13—C8	30.4 (3)	C12—C10—C16—C17	−57.6 (5)
C7—Ru2—C13—C9	152.6 (4)	Ru3—C11—C14—C1	−51.0 (3)
C7—Ru2—C13—C12	−101.7 (3)	Ru3—C11—C14—C9	131.0 (3)
C8—Ru2—C13—C9	122.2 (5)	C4—C11—C14—Ru3	52.2 (3)
C8—Ru2—C13—C12	−132.1 (4)	C4—C11—C14—C1	1.1 (6)
C12—Ru2—C13—C8	132.1 (4)	C4—C11—C14—C9	−176.8 (3)
C12—Ru2—C13—C9	−105.7 (4)	C10—C11—C14—Ru3	−131.1 (3)
C31—Ru2—C13—C8	−110.6 (3)	C10—C11—C14—C1	177.9 (3)
C31—Ru2—C13—C9	11.6 (4)	C10—C11—C14—C9	−0.1 (4)
C31—Ru2—C13—C12	117.3 (3)	Ru2—C12—C13—C8	−54.7 (3)
C32—Ru2—C13—C8	−72.0 (3)	Ru2—C12—C13—C9	129.5 (3)
C32—Ru2—C13—C9	50.3 (4)	C5—C12—C13—Ru2	52.3 (4)
C32—Ru2—C13—C12	156.0 (2)	C5—C12—C13—C8	−2.4 (6)
C33—Ru2—C13—C8	−47.3 (5)	C5—C12—C13—C9	−178.3 (4)
C33—Ru2—C13—C9	74.9 (6)	C10—C12—C13—Ru2	−131.3 (3)
C33—Ru2—C13—C12	−179.4 (4)	C10—C12—C13—C8	174.1 (4)
C34—Ru2—C13—C8	−171.3 (4)	C10—C12—C13—C9	−1.8 (5)
C34—Ru2—C13—C9	−49.1 (7)	C9—C15—C16—C10	−1.1 (4)
C34—Ru2—C13—C12	56.6 (5)	C9—C15—C16—C17	122.6 (3)
C35—Ru2—C13—C8	−148.9 (3)	C18—C15—C16—C10	−124.6 (3)
C35—Ru2—C13—C9	−26.7 (4)	C18—C15—C16—C17	−0.9 (4)
C35—Ru2—C13—C12	79.0 (3)	C9—C15—C18—N1	−96.0 (4)
C5—Ru2—C31—C32	−165.3 (3)	C16—C15—C18—N1	24.2 (4)
C5—Ru2—C31—C35	−47.6 (4)	C10—C16—C17—N1	97.0 (4)
C7—Ru2—C31—C32	45.4 (4)	C15—C16—C17—N1	−22.7 (4)
C7—Ru2—C31—C35	163.2 (3)	N1—C19—C20—Ru1	108.0 (4)
C8—Ru2—C31—C32	81.0 (3)	N1—C19—C20—C21	17.9 (6)
C8—Ru2—C31—C35	−161.3 (3)	N1—C19—C20—C25	−164.7 (4)
C12—Ru2—C31—C32	158.3 (3)	Ru1—C20—C21—C22	54.1 (4)
C12—Ru2—C31—C35	−83.9 (3)	C19—C20—C21—Ru1	124.9 (4)
C13—Ru2—C31—C32	120.1 (3)	C19—C20—C21—C22	179.0 (4)
C13—Ru2—C31—C35	−122.2 (3)	C25—C20—C21—Ru1	−52.6 (4)
C32—Ru2—C31—C35	117.8 (4)	C25—C20—C21—C22	1.5 (7)
C33—Ru2—C31—C32	−37.3 (3)	Ru1—C20—C25—C24	−54.2 (4)
C33—Ru2—C31—C35	80.5 (3)	C19—C20—C25—Ru1	−125.3 (4)
C34—Ru2—C31—C32	−80.0 (3)	C19—C20—C25—C24	−179.4 (4)
C34—Ru2—C31—C35	37.8 (3)	C21—C20—C25—Ru1	52.3 (4)
C35—Ru2—C31—C32	−117.8 (4)	C21—C20—C25—C24	−1.9 (7)
C6—Ru2—C32—C31	173.0 (3)	Ru1—C21—C22—C23	55.7 (4)
C6—Ru2—C32—C33	54.8 (4)	C20—C21—C22—Ru1	−55.6 (4)
C7—Ru2—C32—C31	−152.6 (3)	C20—C21—C22—C23	0.1 (7)
C7—Ru2—C32—C33	89.2 (3)	Ru1—C22—C23—C24	54.3 (4)
C8—Ru2—C32—C31	−113.4 (3)	C21—C22—C23—Ru1	−55.7 (4)
C8—Ru2—C32—C33	128.3 (3)	C21—C22—C23—C24	−1.4 (8)
C12—Ru2—C32—C31	−44.4 (5)	Ru1—C23—C24—C25	54.5 (4)
C12—Ru2—C32—C33	−162.6 (3)	C22—C23—C24—Ru1	−53.4 (4)
C13—Ru2—C32—C31	−76.9 (3)	C22—C23—C24—C25	1.1 (8)
C13—Ru2—C32—C33	164.9 (2)	Ru1—C24—C25—C20	54.8 (4)
C31—Ru2—C32—C33	−118.2 (4)	C23—C24—C25—Ru1	−54.2 (4)
C33—Ru2—C32—C31	118.2 (4)	C23—C24—C25—C20	0.6 (7)
C34—Ru2—C32—C31	80.2 (3)	Ru1—C26—C27—C28	61.5 (3)
C34—Ru2—C32—C33	−38.0 (3)	C30—C26—C27—Ru1	−62.0 (4)
C35—Ru2—C32—C31	37.3 (3)	C30—C26—C27—C28	−0.5 (6)
C35—Ru2—C32—C33	−80.9 (3)	Ru1—C26—C30—C29	−60.7 (4)
C5—Ru2—C33—C32	−178.9 (2)	C27—C26—C30—Ru1	61.5 (4)
C5—Ru2—C33—C34	64.3 (3)	C27—C26—C30—C29	0.8 (6)
C6—Ru2—C33—C32	−143.7 (3)	Ru1—C27—C28—C29	62.2 (3)
C6—Ru2—C33—C34	99.5 (3)	C26—C27—C28—Ru1	−62.2 (4)
C7—Ru2—C33—C32	−104.6 (3)	C26—C27—C28—C29	−0.1 (5)
C7—Ru2—C33—C34	138.6 (3)	Ru1—C28—C29—C30	62.8 (3)
C8—Ru2—C33—C32	−67.9 (3)	C27—C28—C29—Ru1	−62.2 (3)
C8—Ru2—C33—C34	175.3 (2)	C27—C28—C29—C30	0.6 (5)
C13—Ru2—C33—C32	−35.0 (5)	Ru1—C29—C30—C26	61.0 (4)
C13—Ru2—C33—C34	−151.8 (4)	C28—C29—C30—Ru1	−61.8 (3)
C31—Ru2—C33—C32	37.0 (3)	C28—C29—C30—C26	−0.8 (6)
C31—Ru2—C33—C34	−79.8 (3)	Ru2—C31—C32—C33	61.9 (3)
C32—Ru2—C33—C34	−116.8 (4)	C35—C31—C32—Ru2	−61.6 (3)
C34—Ru2—C33—C32	116.8 (4)	C35—C31—C32—C33	0.3 (5)
C35—Ru2—C33—C32	79.2 (3)	Ru2—C31—C35—C34	−61.8 (3)
C35—Ru2—C33—C34	−37.6 (3)	C32—C31—C35—Ru2	61.1 (3)
C5—Ru2—C34—C33	−135.1 (2)	C32—C31—C35—C34	−0.7 (5)
C5—Ru2—C34—C35	107.2 (3)	Ru2—C32—C33—C34	62.7 (3)
C6—Ru2—C34—C33	−95.1 (3)	C31—C32—C33—Ru2	−62.4 (3)
C6—Ru2—C34—C35	147.2 (3)	C31—C32—C33—C34	0.3 (5)
C7—Ru2—C34—C33	−58.5 (3)	Ru2—C33—C34—C35	61.5 (3)
C7—Ru2—C34—C35	−176.2 (3)	C32—C33—C34—Ru2	−62.2 (3)
C12—Ru2—C34—C33	−170.9 (2)	C32—C33—C34—C35	−0.7 (5)
C12—Ru2—C34—C35	71.4 (3)	Ru2—C34—C35—C31	62.0 (3)
C13—Ru2—C34—C33	148.8 (4)	C33—C34—C35—Ru2	−61.1 (3)
C13—Ru2—C34—C35	31.1 (6)	C33—C34—C35—C31	0.8 (5)
C31—Ru2—C34—C33	80.3 (3)	Ru3—C36—C37—C38	−60.9 (6)
C31—Ru2—C34—C35	−37.3 (3)	C40—C36—C37—Ru3	62.1 (5)
C32—Ru2—C34—C33	38.0 (3)	C40—C36—C37—C38	1.2 (9)
C32—Ru2—C34—C35	−79.7 (3)	Ru3—C36—C40—C39	61.3 (6)
C33—Ru2—C34—C35	−117.7 (4)	C37—C36—C40—Ru3	−62.0 (5)
C35—Ru2—C34—C33	117.7 (4)	C37—C36—C40—C39	−0.7 (9)
C5—Ru2—C35—C31	153.3 (2)	Ru3—C37—C38—C39	−62.4 (7)
C5—Ru2—C35—C34	−89.2 (3)	C36—C37—C38—Ru3	61.3 (6)
C6—Ru2—C35—C31	−169.4 (3)	C36—C37—C38—C39	−1.2 (11)
C6—Ru2—C35—C34	−51.9 (4)	Ru3—C38—C39—C40	−61.9 (7)
C8—Ru2—C35—C31	35.7 (5)	C37—C38—C39—Ru3	62.6 (7)
C8—Ru2—C35—C34	153.2 (3)	C37—C38—C39—C40	0.8 (12)
C12—Ru2—C35—C31	114.0 (3)	Ru3—C39—C40—C36	−61.8 (6)
C12—Ru2—C35—C34	−128.5 (3)	C38—C39—C40—Ru3	61.7 (8)
C13—Ru2—C35—C31	75.1 (3)	C38—C39—C40—C36	−0.1 (11)

### Hydrogen-bond geometry (Å, º)

Cg1 is the centroid of the C20–C25 ring and Cg2 is the centroid of the C26–C30 ring.

**Table:**

*D*—H···*A*	*D*—H	H···*A*	*D*···*A*	*D*—H···*A*
C2—H2···O2^i^	0.95	2.56	3.188 (8)	124
C3—H3···O2^i^	0.95	2.58	3.197 (7)	123
C7—H7···F7^ii^	0.95	2.50	3.359 (5)	150
C8—H8···F9^iii^	0.95	2.42	3.302 (6)	155
C16—H16···O1	1.00	2.46	3.240 (6)	134
C18—H18*A*···F12^iii^	0.99	2.52	3.398 (5)	147
C22—H22···F11^iv^	0.95	2.43	3.091 (6)	127
C27—H27···O2^v^	0.95	2.50	3.141 (9)	125
C29—H29···F6^ii^	0.95	2.48	3.155 (7)	128
C43—H43*C*···F4^iv^	0.98	2.31	3.287 (9)	176
C46′—H46*D*···*Cg*1	0.98	2.79	3.63 (5)	160
C32—H32···*Cg*2^vi^	0.95	2.43	3.669 (5)	136

Symmetry codes: (i) *x*−1, *y*, *z*−1; (ii) −*x*+1/2, *y*+1/2, −*z*+3/2; (iii) *x*−1/2, −*y*+1/2, *z*−1/2; (iv) *x*+1/2, −*y*+1/2, *z*−1/2; (v) *x*−1, *y*, *z*; (vi) −*x*, −*y*+1, −*z*+1.
